# How and when EEG reflects changes in neuronal connectivity due to time awake

**DOI:** 10.1016/j.isci.2023.107138

**Published:** 2023-06-19

**Authors:** Sophia Snipes, Elias Meier, Sarah Nadine Meissner, Hans-Peter Landolt, Reto Huber

**Affiliations:** 1 Child Development Center, University Children’s Hospital Zürich, University of Zürich, 8032 Zürich, Switzerland; 2 Neural Control of Movement Lab, Department of Health Sciences and Technology, ETH Zürich, 8092 Zürich, Switzerland; 3 Institute of Pharmacology and Toxicology, University of Zürich, Zürich, 8057 Zürich, Switzerland; 4 Sleep & Health Zürich, University of Zürich, Zürich, 8006 Zürich, Switzerland; 5 Department of Child and Adolescent Psychiatry and Psychotherapy, Psychiatric Hospital, University of Zürich, 8008 Zürich, Switzerland

**Keywords:** Neuroscience, Behavioral neuroscience, Cognitive neuroscience

## Abstract

Being awake means forming new memories, primarily by strengthening neuronal synapses. The increase in synaptic strength results in increasing neuronal synchronicity, which should result in higher amplitude electroencephalography (EEG) oscillations. This is observed for slow waves during sleep but has not been found for wake oscillations. We hypothesized that this was due to a limitation of spectral power analysis, which does not distinguish between changes in amplitudes from changes in number of occurrences of oscillations. By using cycle-by-cycle analysis instead, we found that theta and alpha oscillation amplitudes increase as much as 30% following 24 h of extended wake. These increases were interrupted during the wake maintenance zone (WMZ), a window just before bedtime when it is difficult to fall asleep. We found that pupil diameter increased during this window, suggesting the ascending arousal system is responsible. In conclusion, wake oscillation amplitudes reflect increased synaptic strength, except during the WMZ.

## Highlights

• Wake oscillation amplitudes increase with time awake, reflecting sleep homeostasis
• Oscillation quantities change independently, so spectral power is unreliable
• The brain desynchronizes during the wake maintenance zone, and pupil sizes increase
• This zone may be a unique state of alertness, specific to humans

## Graphical abstract

**Figure:**
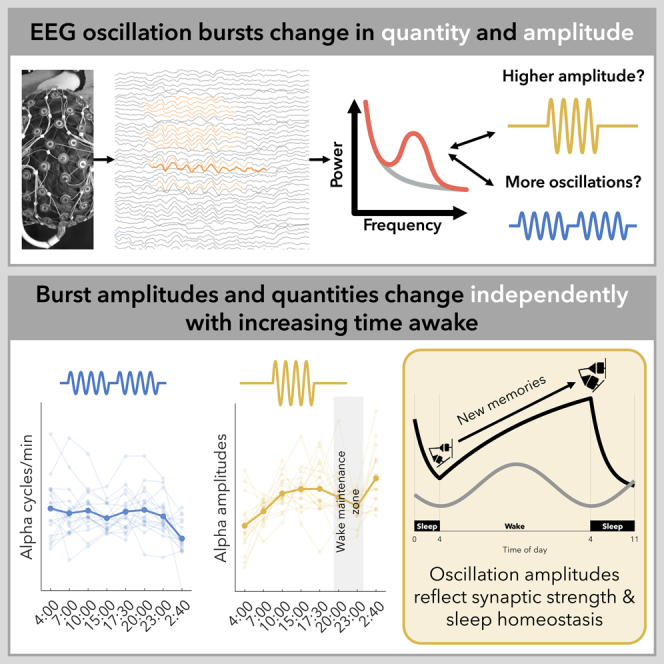

## Introduction

Good sleep is essential for daily functioning and overall quality of life. The reason we need sleep is so that physiological systems used during the day have a dedicated period to rest and conduct structural maintenance,^1^ clear metabolic by-products,^2^^,^^3^ restore overall functioning to baseline levels,^4^^,^^5^ and more. This means that we accumulate sleep need with time awake and can only restore balance following sleep, a process referred to as *sleep homeostasis.* Also important is the timing of sleep, controlled by a 24 h *circadian rhythm* which allows independent systems across the body and brain to synchronize their recovery to the external world’s day/night cycle and thus optimize overall performance.^6^^,^^7^ These homeostatic and circadian fluctuations make up the two-process model of sleep.^8^

**Figure 1:**
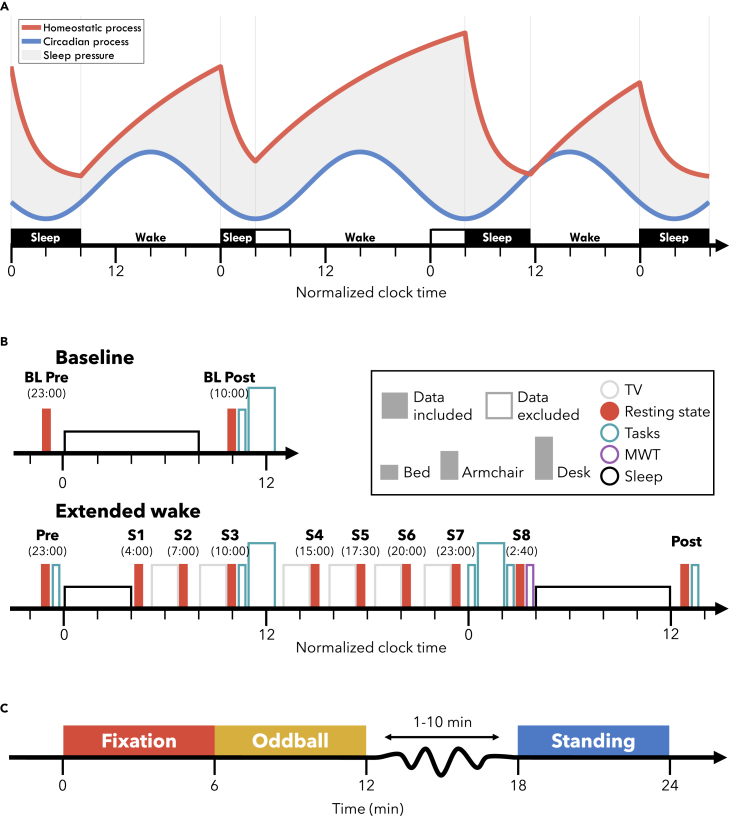
Experiment design (A) The two-process model during a 4/24 extended wake schedule. The red line reflects the homeostatic process, building sleep pressure monotonically with wake and dissipating during sleep. The blue line reflects the circadian process, peaking in the middle of the day and at its lowest in the middle of the night, independent of actual sleep and wake behavior. The shaded area reflects the resulting sleep pressure from combining these two processes. Black filled blocks indicate when participants actually slept, whereas the outline indicates the window in which they would have slept according to their circadian rhythm. (B) Experiment schedule. Each block indicates an EEG recording session. Filled blocks indicate data analyzed in this paper. Color indicates the activity participants engaged in: gray, watching TV; red, the resting state recordings in C; teal, task blocks analyzed in Snipes et al.^70^; purple, the MWT; black, sleep. The height of each block indicates the condition in which data were collected: short, lying in bed; medium, seated in a comfortable armchair with foot and backrest/standing; tall, seated at a desk. Brief empty spaces indicate transition periods allowing for delays. Six longer breaks were included prior to each TV block in which participants were provided with meals. Circadian time was normalized across participants to their habitual bedtime. Participants at baseline and during the recovery night were free to wake up when they wished, and at the beginning of the extended wake period they were woken up after 4 h of sleep. (C) Timeline for the resting state recordings. Each condition was 6 min, and always done in the depicted order. Between the Oddball and Standing, a questionnaire was conducted which took a variable amount of time, followed by moving the participant from the armchair to standing. Abbreviations: BL, baseline; MWT, maintenance of wakefulness task.

The homeostatic process of the two-process model in particular was designed to explain the notable changes in sleep slow waves, electroencephalographic (EEG) oscillations between 0.5 and 4 Hz that characterize NREM sleep (non rapid-eye-movement sleep, i.e. stages 2 & 3).^9^ Slow wave activity decreases exponentially during NREM sleep, reflecting homeostatic sleep pressure dissipation. Vice versa, slow wave activity at the beginning of sleep depends on the duration of prior wake, following an increasing saturating exponential function.^10^^,^^11^ This means that the buildup in sleep need is steepest during the initial hours of wake, then gradually saturates with additional time awake (Figure 1A).

A possible explanation for this increase in slow wave activity with prior wake is that wakefulness progressively increases neuronal synaptic strength when forming new memories, which then requires sleep to restore overall synaptic balance. This is referred to as *synaptic homeostasis*, as described by the synaptic homeostasis hypothesis.^12^ In essence, learning and acquiring memories requires changes to the brain in the form of strengthened synaptic connections between utilized neurons. Increased synaptic strength increases overall connectivity, which leads to increased synchronicity across the brain. This increased synchronicity between neurons will result in more synchronized oscillations in the surface EEG, detected as oscillations with larger amplitudes and steeper slopes as time awake increases. The hypothesis then predicts that sleep, when inputs cease and new memories are no longer acquired, is the only time when synaptic balance can be re-normalized to baseline levels.^12^^,^^13^ Using computational models,^14^ animal sleep data,^15^ and human sleep data,^16^ the proponents of the synaptic homeostasis hypothesis demonstrated how decreasing synaptic strength across sleep results in the decrease of slow wave amplitudes and slopes.

While the combined models of sleep homeostasis and synaptic homeostasis can explain changes in slow wave activity across sleep, they do not likewise explain changes in wake oscillations. Human wake EEG is predominantly characterized by alpha oscillations (8–12 Hz) and to a lesser extent theta oscillations (4–8 Hz), often measured as power in the frequency domain. Theoretically, the increased connectivity with time spent awake should affect these oscillations along a similar increasing saturating exponential function as for slow waves in sleep. However, while theta power does increase with sleep deprivation, the effect is rather linear.^17^ Furthermore alpha power actually decreases.^18^^,^^19^

In addition to neither oscillation following a homeostatic trajectory, both are also affected by circadian rhythmicity,^17^^,^^18^^,^^19^^,^^20^ further masking potential homeostatic effects. Alpha activity fluctuates in phase with core body temperature, a reliable circadian marker peaking in the middle of the day and lowest in the middle of the night.^18^^,^^21^ Instead, theta activity is lowest in the evening,^18^ corresponding to the wake maintenance zone (WMZ).^22^^,^^23^ The WMZ, more dramatically known as the “forbidden sleep zone,” is a circadian window of 2–4 h just prior to melatonin onset in which sleep becomes exceptionally difficult.^24^ During the WMZ, sleep onset latencies substantially increase even during extensive sleep deprivation,^25^^,^^26^ subjective sleepiness decreases, and behavioral performance improves.^23^^,^^27^^,^^28^^,^^29^ The timing of the WMZ is not reflected in traditional circadian markers such as melatonin levels or core body temperature, it has not been reported in any animal models to our knowledge, and is not represented in the classic two-process model of sleep.

Therefore, while increasing synaptic strength would have predicted an increase in both theta and alpha power with time spent awake, in practice neither oscillation strictly reflects this buildup in homeostatic pressure, and they are further synchronized to different circadian phases. However, the fact that wake oscillations do not reflect sleep homeostasis may be due to a limitation of spectral power analyses. “Power” refers to the amount of energy in a frequency band, and is typically calculated using some variant of the Fast Fourier Transform.^30^ Once a time-series signal has been transformed into the frequency domain, power values are averaged or summed within a frequency range of interest, and this is the power for that band. While this is a simple and generally effective measure for quantifying oscillatory activity, it is simultaneously affected by the *quantity* of oscillations present in the signal and their *amplitude,* as well as broadband changes in the entire spectrum.^31^

The synaptic homeostasis hypothesis predicts that an increase in synaptic connectivity results in an increase in oscillatory amplitudes; this does not need to have any bearing on the number of oscillations that actually occur. It is therefore possible that non-homeostatic factors such as the WMZ could independently affect the quantity of oscillations, whereas time spent awake more specifically affects their amplitude. When both oscillation amplitudes and quantities change independently across wake recordings, the resulting power values will reflect some undifferentiated mix between the two. By separating these contributions, we may have a specific marker of homeostatic sleep pressure during wake. Not only would this provide supporting evidence for the hypothesis that sleep homeostasis is linked to synaptic plasticity, but also provide a marker for sleep pressure more easily acquired than slow wave activity during sleep.

We therefore wished to determine whether the circadian and homeostatic influences on theta and alpha oscillations could be dissociated in resting wake EEG by separately measuring changes in amplitude and changes in quantities of oscillations. Eighteen young healthy adults participated in a 4/24 extended wake paradigm (Figure 1A), in which they slept the first 4 h of the night and were then kept awake for 24 h with repeated resting state recordings (Figure 1B), while measuring high-density EEG. We conducted cycle-by-cycle analysis to identify bursts of oscillations in the theta and alpha range, a method which identifies oscillations based on the morphology of the EEG signal rather than relying on power and amplitude thresholds (for an in-depth explanation, see STAR Methods: method details: EEG burst detection).^32^ We then looked at changes in the mean amplitude of bursts and the average number of cycles (i.e., oscillations present in a burst) per minute for each band. Our prediction was that both theta and alpha amplitudes would follow an increasing saturating exponential across extended wake and show decreases following sleep. At the same time, the decrease in alpha power with time awake should be explained by a decrease in the overall number of alpha oscillations. Likewise, circadian changes such as the decrease in theta during the WMZ should be reflected in decreases in the number of bursts.

To independently monitor changes in alertness across the extended wake period, we also recorded pupillometry with infrared cameras. Pupil diameter and pupil responses to salient stimuli have been linked to alertness-promoting activity in the locus coeruleus (LC),^33^^,^^34^^,^^35^ as well as other interconnected nuclei in the brainstem and forebrain that make up the ascending arousal system (AAS).^36^^,^^37^ While there are still many open questions about the link between pupil size and sleep/wake promoting nuclei, the relationship between any of these signals and EEG could help better explore the underlying mechanisms driving the changes in oscillatory activity across extended wake. In short, while the two-process model and the synaptic homeostasis hypothesis provide specific predictions about wake EEG amplitudes, with pupillometry we hoped to provide possible explanations for changes in oscillatory occurrences.

## Results

We recorded EEG, pupillometry, and questionnaire data from participants performing 3 wake resting state recordings for 6 min each (Figure 1C). The first was a standard condition, *Fixation*, in which participants were seated in a comfortable armchair and had to gaze at a fixation point ∼3 m away. The second was an active auditory *Oddball*, in which tones were presented randomly and participants had to push a button after “oddball” (i.e. deviant) tones. Afterward, participants filled out a questionnaire and then got up for the final recording of *Standing* with eyes closed. They were asked to stand for this condition because during sleep deprivation participants quickly fall asleep with eyes closed. These rest recordings were conducted 12 times: before and after each of the 3 nights of sleep, and 6 more times throughout the extended wake period approximately 3 h apart, for a total of 8 recordings across extended wake (Figure 1B). The primary focus of this study was the Fixation condition, used in previous studies. The auditory Oddball was included as an exploratory condition to evaluate changes in pupillometry with time awake, especially responses to target tones which are thought to reflect activity in the LC.^35^ The EEG of both the Oddball and the Standing with eyes-closed conditions were investigated in order to establish the sensitivity to homeostatic effects of other conditions.

**Figure 2:**
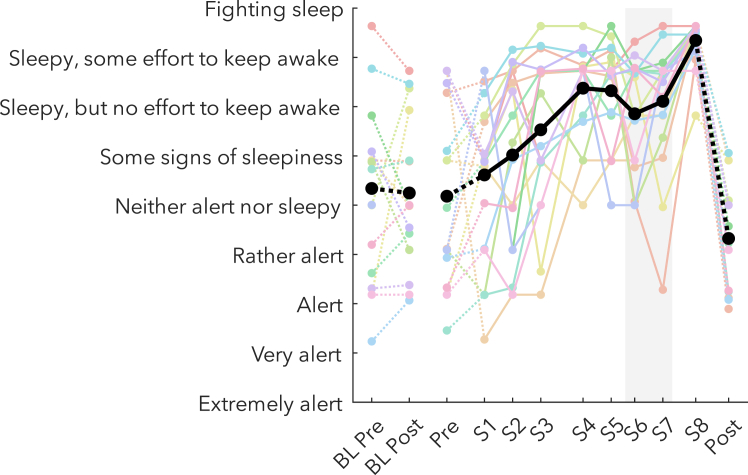
Subjective sleepiness Sleepiness was measured on a continuous visual-analog adaptation of the KSS, using the original labels as markers (y axis). The thick black line indicates the group average, and thin colored lines are datapoints of individual participants. Solid lines connect sessions during the same-day extended wake period, and dashed lines indicate changes across sleep. S1–S8 are spaced out relative to the time they occurred within the 24 h wake period (Figure 1B). The shaded gray area indicates the WMZ. Acronyms: BL, baseline; KSS, Karolinska Sleepiness Scale; WMZ, wake maintenance zone.

For every outcome measure, we conducted paired t-tests on the overnight changes of the baseline night (BL pre vs. BL post), the extended wake changes (S1 vs. S8) and the changes during the WMZ (S5/S8 vs. S6/S7), the timing of which could be independently determined through changes in subjective sleepiness (Figure 2). All t-values, degrees of freedom, p values, and Hedge’s g effect sizes are provided together in Table 1. Throughout the text, only the corresponding t-values will be reported, unless either effect sizes or p values are specifically of interest (e.g. when trending).

**Table 1:** Statistics results

Outcome measure	Condition	Overnight baseline	Extended wake	WMZ
Sleepiness	–	t_(15)_ = −0.21, p = 0.833, g = −0.06	t_(16)_ = 7.36, **p < 0.001**, g = 2.61	t_(16)_ = −3.36, **p = 0.004**, g = −1.13
Theta power	Fixation	t_(17)_ = −4.80, **p < 0.001**, g = −0.83	t_(16)_ = 9.66**, p < 0.001**, g = 2.99	t_(16)_ = −5.96, **p < 0.001**, g = −1.78
Theta power	Oddball	t_(17)_ = −3.19, **p = 0.005**, g = −0.70	t_(16)_ = 10.41, **p < 0.001**, g = 3.76	t_(16)_ = −6.92, **p < 0.001**, g = −2.17
Theta power	Standing	t_(16)_ = −0.57, p = 0.574, g = −0.14	t_(16)_ = 5.97, **p < 0.001**, g = 2.26	t_(16)_ = −5.19, **p < 0.001**, g = −1.21
Alpha power	Fixation	t_(17)_ = 1.75, p = 0.098, g = 0.26	t_(16)_ = 3.03, **p = 0.008**, g = 1.07	t_(16)_ = −2.86, **p = 0.011**, g = −0.63
Alpha power	Oddball	t_(17)_ = 0.82, p = 0.421, g = 0.13	t_(16)_ = 3.89, **p = 0.001**, g = 1.32	t_(16)_ = −3.61, **p = 0.002**, g = −0.89
Alpha power	Standing	t_(16)_ = 2.57, **p = 0.020**, g = 0.71	t_(16)_ = −1.44, p = 0.168, g = −0.49	t_(16)_ = −1.13, p = 0.276, g = −0.16
Theta burst amplitude	Fixation	t_(15)_ = −2.06, p = 0.057, g = −0.74	t_(16)_ = 6.71, **p < 0.001**, g = 2.40	t_(16)_ = −3.19, **p = 0.006**, g = −0.70
Theta burst amplitude	Oddball	t_(16)_ = −1.99, p = 0.064, g = −0.47	t_(15)_ = 6.92, **p < 0.001**, g = 2.51	t_(16)_ = −4.78, **p < 0.001**, g = −1.50
Theta burst amplitude	Standing	t_(16)_ = −2.55, **p = 0.022**, g = −0.55	t_(16)_ = 2.15, **p = 0.047**, g = 0.80	t_(16)_ = −0.46, p = 0.655, g = −0.10
Alpha burst amplitude	Fixation	t_(17)_ = −5.55**, p < 0.001**, g = −0.75	t_(16)_ = 4.49, **p < 0.001**, g = 1.65	t_(16)_ = −3.71, **p = 0.002**, g = −0.97
Alpha burst amplitude	Oddball	t_(17)_ = −2.00, p = 0.061, g = −0.43	t_(16)_ = 7.52, **p < 0.001**, g = 2.18	t_(16)_ = −6.47, **p < 0.001**, g = −1.58
Alpha burst amplitude	Standing	t_(16)_ = 0.14, p = 0.889, g = 0.04	t_(16)_ = 0.17, p = 0.870, g = 0.06	t_(16)_ = −2.22, **p = 0.041**, g = −0.32
Theta burst cycles/min	Fixation	t_(17)_ = 1.08, p = 0.295, g = 0.34	t_(16)_ = 5.57, **p < 0.001**, g = 2.00	t_(16)_ = −1.77, p = 0.095, g = −0.58
Theta burst cycles/min	Oddball	t_(17)_ = 1.25, p = 0.227, g = 0.30	t_(16)_ = 7.80, **p < 0.001**, g = 2.52	t_(16)_ = −4.03, **p = 0.001**, g = −1.24
Theta burst cycles/min	Standing	t_(16)_ = 0.67, p = 0.513, g = 0.24	t_(16)_ = 4.68, **p < 0.001**, g = 1.70	t_(16)_ = −5.02, **p < 0.001**, g = −1.66
Alpha burst cycles/min	Fixation	t_(17)_ = 3.32, **p = 0.004**, g = 0.96	t_(16)_ = −2.87, **p = 0.011**, g = −1.13	t_(16)_ = 1.98, p = 0.065, g = 0.67
Alpha burst cycles/min	Oddball	t_(17)_ = 2.63, **p = 0.017**, g = 0.77	t_(16)_ = −3.31, **p = 0.004**, g = −1.37	t_(16)_ = 2.51, **p = 0.023**, g = 0.48
Alpha burst cycles/min	Standing	t_(16)_ = 3.63, **p = 0.002**, g = 0.89	t_(16)_ = −6.17, **p < 0.001**, g = −1.73	t_(16)_ = 2.91, **p = 0.010**, g = 0.56
Pupil diameter (mean)	Fixation	t_(13)_ = 0.39, p = 0.699, g = 0.13	t_(16)_ = −3.78, **p = 0.002**, g = −1.22	t_(15)_ = 4.65, **p < 0.001**, g = 1.26
Pupil diameter (mean)	Oddball	t_(12)_ = −1.79, p = 0.099, g = −0.47	t_(12)_ = −1.39, p = 0.189, g = −0.59	t_(12)_ = 2.17, p = 0.051, g = 0.64
Pupil diameter (SD)	Fixation	t_(13)_ = −3.76, **p = 0.002**, g = −0.98	t_(16)_ = 3.52, **p = 0.003**, g = 1.25	t_(15)_ = −1.69, p = 0.111, g = −0.65
Pupil diameter (SD)	Oddball	t_(12)_ = −3.66, **p = 0.003**, g = −1.22	t_(12)_ = 4.74, **p < 0.001**, g = 1.56	t_(12)_ = −2.58, **p = 0.024**, g = −0.90
Pupil oddball response	Oddball	t_(9)_ = 0.26, p = 0.802, g = 0.07	t_(11)_ = −1.58, p = 0.143, g = −0.55	t_(9)_ = 2.08, p = 0.067, g = 0.74
Blink rate	Fixation	t_(13)_ = −1.32, p = 0.209, g = −0.39	t_(16)_ = 0.62, p = 0.545, g = 0.22	t_(15)_ = −1.25, p = 0.229, g = −0.42
Blink rate	Oddball	t_(13)_ = −0.02, p = 0.987, g = −0.01	t_(13)_ = 4.37, **p = 0.001**, g = 1.44	t_(15)_ = −0.21, p = 0.839, g = −0.08
Ocular microsleeps (%)	Fixation	t_(13)_ = −0.97, p = 0.350, g = −0.32	t_(16)_ = 4.81, **p < 0.001**, g = 1.56	t_(15)_ = −4.85, **p < 0.001**, g = −1.68
Ocular microsleeps (%)	Oddball	t_(13)_ = −1.32, p = 0.210, g = −0.47	t_(13)_ = 4.18, **p = 0.001**, g = 1.41	t_(15)_ = −6.28, **p < 0.001**, g = −2.16

Paired t-tests were conducted to determine overnight changes at baseline (BL Pre vs. BL Post), changes across 24 h of extended wake (S1 vs. S8), and deviations from the wake trajectories during the WMZ (S5&S8 vs. S6&S7). All values were z-scored for each participant, pooling sessions, and conditions. Power values were z-scored separately for each frequency prior to being averaged into bands, and pupil oddball responses were z-scored also across timepoints prior to measuring the average response. Degrees of freedom are specified in the subscript of t-values and reflect the sample size for each comparison (N = DF + 1). Effect sizes are provided as Hedge’s g values. All statistics are with α = 5%, significant p values are in bold. There is no correction for multiple comparisons, given that the hypothesis being tested only applied to extended wake changes in the Fixation condition, and all other tests were either exploratory or confirmatory (see STAR Methods). Acronyms: WMZ, wake maintenance zone; BL, baseline; SD, standard deviation.

### Changes in theta power but not alpha power replicate previous results

Before investigating oscillatory burst activity, we first determined whether our 4/24 experimental paradigm replicated findings of previous studies showing both circadian and homeostatic changes in theta and alpha power. We expected an increase in theta and a decrease in alpha with increasing time awake, as well as a dip in theta during the WMZ, and a peak in alpha in the middle of the day.^18^ Power spectral density was calculated using Welch’s method for every channel during each recording. These values were z-scored separately for each frequency, pooling channels, conditions, and sessions. Z-scored power values were then averaged across channels, and then averaged within the theta and alpha bands.

**Figure 3:**
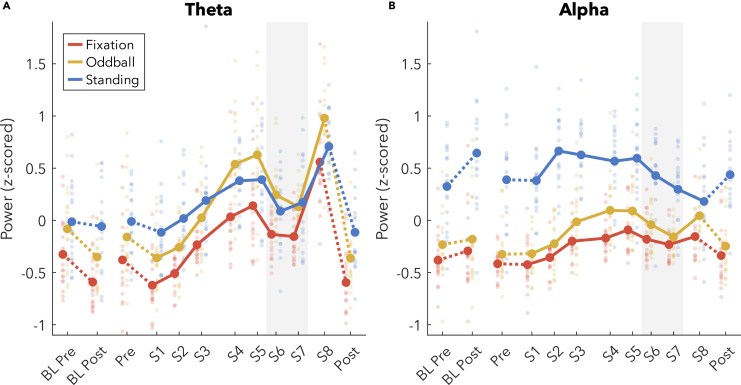
Power band changes, z-scored (A and B) (A) Theta power (4–8 Hz) and (B) alpha power (8–12 Hz). Thick lines indicate group averages for each condition (as different colors) across sessions (x axis). Solid lines connect sessions during the same-day extended wake period, and dashed lines indicate changes across sleep. S1–S8 are spaced out relative to the time they occurred within the 24 h wake period (Figure 1B). Dots reflect individual participants’ datapoints. The shaded gray area indicates the WMZ. Power spectral density values were first z-scored for each frequency pooling channels, sessions, and conditions. All channels were included in the average except edge channels: 48, 63, 68, 73, 81, 88, 94, 99, and 119. Finally, z-scored values within each band range were averaged. Acronyms: BL, baseline; WMZ, wake maintenance zone.

Changes in theta power are plotted in Figure 3A. After the baseline night, there was a significant decrease in theta power for the Fixation (t_(17)_ = −4.80) and Oddball recordings (t_(17)_ = −3.19), but no change during Standing with eyes closed (t_(16)_ = −0.57). Across extended wake there was a substantial increase in theta power in all conditions (Fixation, t_(16)_ = 9.66; Oddball, t_(16)_ = 10.41; Standing, t_(16)_ = 5.97). During the WMZ, all conditions showed very large and significant decreases in theta power (Fixation, t_(16)_ = −5.96; Oddball, t_(16)_ = −6.92; Standing, t_(16)_ = −5.19).

Changes in alpha power are plotted in Figure 3B. After the baseline night, alpha power showed a trend increase for Fixation (t_(17)_ = 1.75, p = 0.098), no change for Oddball (t_(17)_ = 0.82), and a significant increase during Standing (t_(16)_ = 2.57). Across extended wake, alpha actually increased for Fixation (t_(16)_ = 3.03) and Oddball (t_(16)_ = 3.89) and showed no significant change during Standing, although on average decreased (t_(16)_ = −1.44). A significant dip in alpha was present during the WMZ in the Fixation condition (t_(16)_ = −2.86) and even more prominent in the Oddball (t_(16)_ = −3.61).

Given the discrepancy with previous results that found decreases in alpha with sleep deprivation,^18^^,^^19^ we inspected the spectrograms of z-scored power to determine whether some other factor was contributing to the increase in alpha in our data (Figure S1). We found broadband increases in power with time awake, as well as increases in theta and beta power extending into the alpha range. Furthermore, unlike previous experiments, our 4/24 design is such that S1 and S8 were during the lowest circadian points for alpha power. Therefore, the broadband and neighboring band effects may have had a stronger influence on final alpha power compared to previous studies, thus explaining the discrepancy.

### Oscillation amplitudes increase with extended wake independently from quantities, but decrease during the WMZ

**Figure 4:**
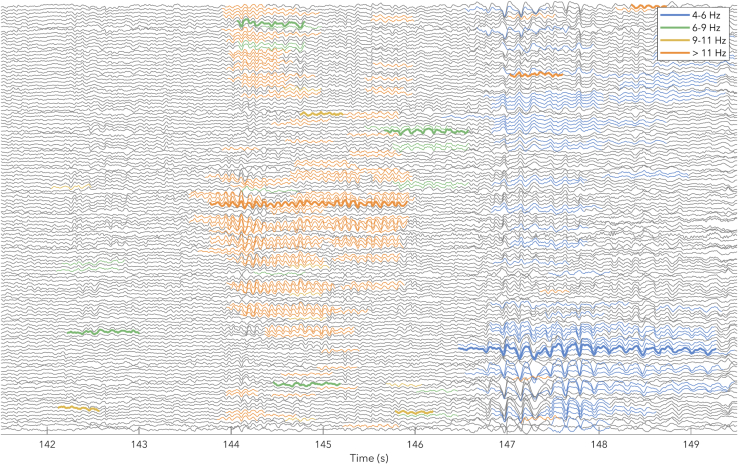
Example of detected bursts 10 s of data from P15 Fixation (see Figure S3A). EEG data traces are in gray. Thick colored lines indicate the “reference” burst, the longest among temporally overlapping bursts in the same channel. Thin colored lines indicate overlapping bursts across channels considered to be the “same” burst as the reference. These were associated with the reference because mean frequencies were within 1 Hz of each other. Different colors represent different frequency bands.

Cycle-by-cycle analysis was used to identify bursts between 2 and 14 Hz (a schematic of the algorithm is provided in figure in STAR Methods). Figure 4 provides an example of the EEG and burst detection during S8. The detected bursts were then split into theta (mean frequency between 4 and 8 Hz), and alpha (8 and 12 Hz). Oscillation amplitudes were quantified as the average negative-to-positive peak voltage for all the cycles involved in a burst. The “number” of oscillations was quantified as the number of cycles per minute.

**Figure 5:**
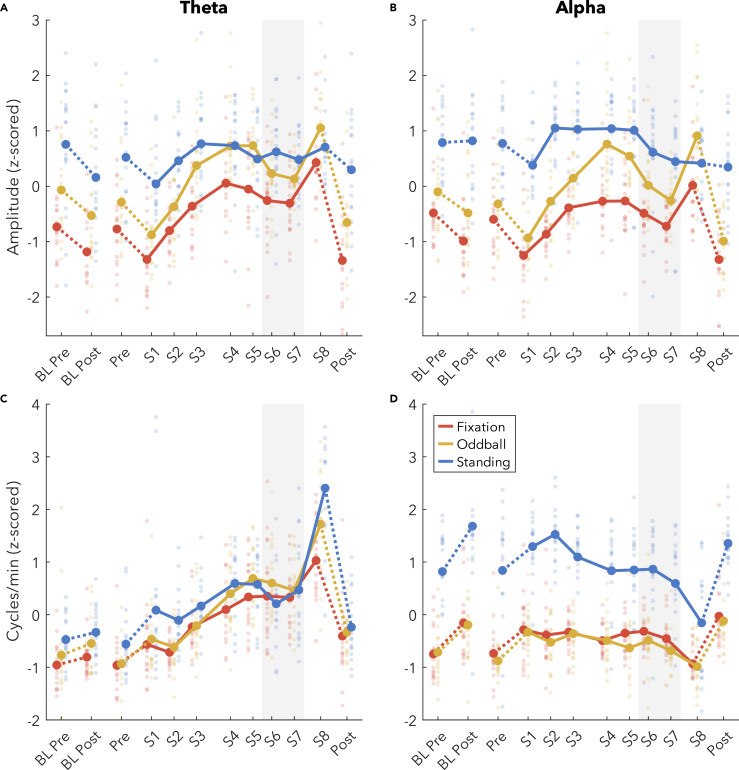
Burst changes in amplitude and quantity, z-scored (A–D) (A) Average theta burst amplitudes, (B) alpha burst amplitudes, (C) number of theta cycles per minute, (D) alpha cycles per minute. Thick lines indicate group averages for each condition across sessions (x axis), with color indicating condition. Solid lines connect sessions during the same-day extended wake period, and dashed lines indicate changes across sleep. S1–S8 are spaced out relative to the time they occurred within the 24 h wake period (Figure 1B). Dots reflect individual participants’ datapoints. The shaded gray area indicates the WMZ. All values are z-scored within each figure, such that sessions and conditions were pooled. Acronyms: BL, baseline; WMZ, wake maintenance zone.

Figure 5 plots the change in theta and alpha bursts by average amplitude (Figures 5A and 5B), and cycles per minute (Figures 5C and 5D). Amplitudes tended to decrease after baseline sleep for theta (Fixation: t_(15)_ = −2.06, p = 0.057; Oddball: t_(16)_ = −1.99, p = 0.064; Standing: t_(16)_ = −2.55, p = 0.022). Amplitudes significantly decreased after sleep for alpha Fixation (t_(17)_ = −5.55), were trending for Oddball (t_(17)_ = −2.00, p = 0.061), and showed no change during Standing (t_(16)_ = 0.14). During extended wake, amplitudes increased substantially for both theta and alpha in the Fixation (theta t_(16)_ = 6.71; alpha t_(16)_ = 4.49) and Oddball conditions (theta t_(15)_ = 6.92; alpha t_(16)_ = 7.52). Theta amplitudes increased during wake in Standing (t_(16)_ = 2.15, p = 0.047) but no change was observed in alpha amplitudes during Standing (t_(16)_ = 0.17). The trajectory of the increase in amplitudes for both theta and alpha, and Fixation and Oddball, approximated that of an increasing saturating exponential function, with steeper increases at the beginning (S1 to S3 theta Fixation increased by 19% [interquartile range (IQR): 7, 27], theta Oddball 19% [10, 26]; alpha Fixation 14% [5, 19], alpha Oddball 14% [7, 20]) compared to the end of the wake period (S1 to S8 theta Fixation 31% [10, 42], theta Oddball 29% [20, 35]; alpha Fixation 20% [9, 28]; alpha Oddball 27% [13, 34]). Theta Standing amplitudes only increased across the first 6 h (S1 to S3: Standing 11% [0, 16]; S1 to S8: Standing 11% [-2, 22]), and alpha increased from S1 to S2, then decreased from S5 to S6. Against our expectations, however, both theta and alpha showed a robust decrease in amplitude during the WMZ for both Fixation (theta t_(16)_ = −3.19; alpha t_(16)_ = −3.71) and Oddball (theta t_(16)_ = −4.78; alpha t_(16)_ = −6.47).

Changes across baseline sleep in cycles per minute went in the opposite direction from amplitudes: theta quantities on average increased although this was not significant (Fixation t_(17)_ = 1.08; Oddball t_(17)_ = 1.25; Standing t_(16)_ = 0.67) and alpha significantly increased (Fixation t_(17)_ = 3.32; Oddball t_(17)_ = 2.63; Standing t_(16)_ = 3.63). During extended wake, theta quantities significantly increased in all conditions along a mostly linear trajectory (Fixation t_(16)_ = 5.57; Oddball t_(16)_ = 7.80; Standing t_(16)_ = 4.68), whereas alpha quantities decreased (Fixation t_(16)_ = −2.87; Oddball t_(16)_ = −3.31; Standing t_(16)_ = −6.17), primarily during S8. During S1, theta occupied on average 8% (1, 8) of the Fixation recording (Oddball: 10% [1, 13]; Standing: 12% [2, 16]) and more than this doubled to 22% (4, 34) during S8 (Oddball: 25% [7, 37]; Standing: 27% [8, 33]). Instead, alpha occupied 54% (24, 78) of the Fixation recording during S1 (Oddball: 54% [27, 72]; Standing: 80% [59, 96]), which decreased to 42% (26, 58)] during S8 (Oddball: 41% [26, 57]; Standing: 56% [35, 78]).

Theta and alpha cycles per minute were significantly affected by the WMZ in opposite directions during the Standing (theta t_(16)_ = −5.02; alpha t_(16)_ = 2.51) and Oddball conditions (theta t_(16)_ = −4.03; alpha t_(16)_ = 2.91) but trending in Fixation (theta t_(16)_ = −1.77, p = 0.095; alpha t_(16)_ = 1.98, p = 0.065), such that theta quantities decreased relative to the overall trajectory, and alpha increased (or did not decrease along the expected trajectory).

**Figure 6:**
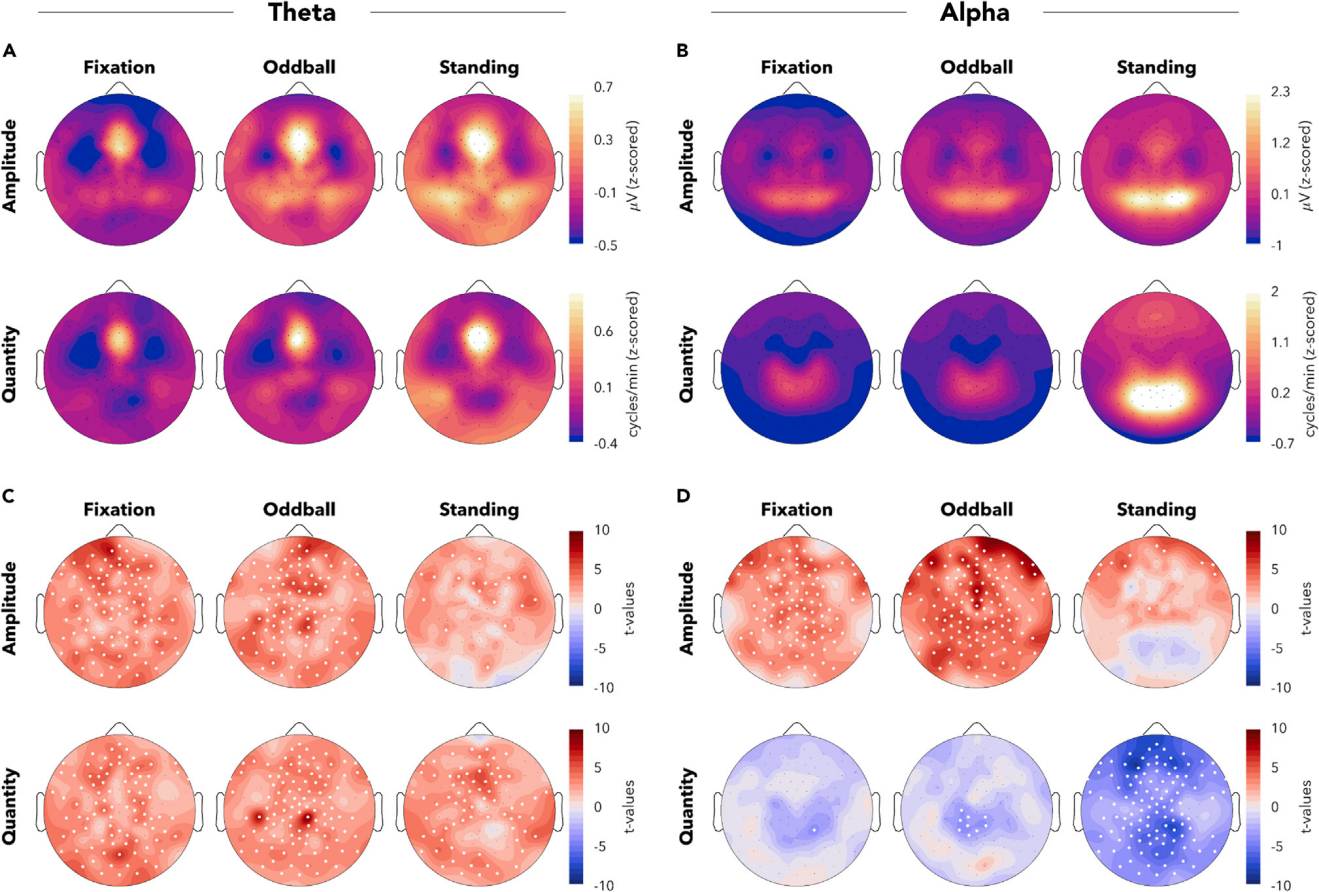
Topographic distribution of burst amplitudes and quantities (A and B) Amplitudes (top row) and cycles per minute (bottom row) for theta (A) and alpha bursts (B) across 123 channels for each condition, z-scored and averaged across all sessions. Lighter colors indicate higher amplitudes/quantities. (C and D) Change in amplitudes and cycles/min from S1 to S8 for theta (C) and alpha bursts (D) represented as t-values, such that red indicates an increase with time awake. White dots indicate channels for which the difference was statistically significant (p < 0.05, N = 17) based on paired t-tests, with false discovery rate correction.

To determine whether oscillation amplitudes and quantities originated from the same areas, we inspected the mean distribution of amplitudes and cycles per minute for theta and alpha bursts across the 123 channels, pooling sessions (Figures 6A and 6B). To determine whether the changes observed in Figure 5 were spatially dependent, we performed paired t-tests between S1 and S8 for each channel, with false-discovery rate (FDR) correction (Figures 6C and 6D).

For all three conditions, theta bursts were located primarily in frontal-midline channels, which also generated the largest amplitudes (Figure 6A). The increases observed with extended wake were widespread although somewhat patchy for both amplitudes and cycles per minute (Figure 6C) and were not limited to the main frontal-midline sources of theta. Alpha amplitudes and cycles per minute were instead spatially dissociated (Figure 6B), with high amplitudes originating more occipitally, and high quantities originating more centro-parietally. While the increase in alpha amplitudes in the Fixation and Oddball were similarly widespread as in theta, during the Standing condition the increase was only frontal (Figure 6D, top row). Instead, the decrease in alpha quantities was localized to the centro-parietal regions in Fixation and Oddball, with more widespread decreases in Standing (Figure 6D, bottom row).

### Pupil diameter means and standard deviations change during the WMZ, while pupil responses to oddball tones do not

**Figure 7:**
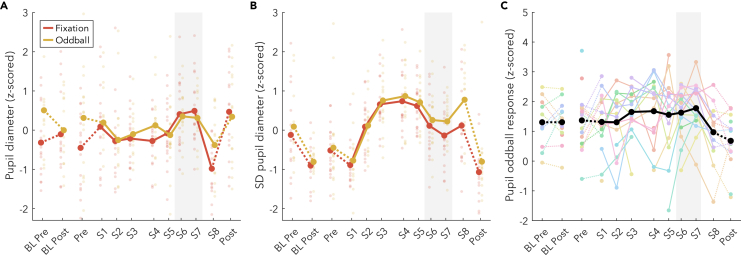
Pupil diameter (A and B) (A) Mean diameter and (B) standard deviations across the 6 min recordings. Thick lines indicate group averages for each condition across sessions (x axis). Solid lines connect sessions during the same-day extended wake period, and dashed lines indicate changes across sleep. Color indicates condition, dots reflect individual participants’ datapoints. S1–S8 are spaced out relative to the time they occurred within the 24 h wake period (Figure 1B). The shaded gray area indicates the WMZ. All values are z-scored within each figure, such that sessions and conditions were pooled. (C) Oddball pupil diameter response to target tones relative to standard tones from 0.5 to 2 s after tone onset. Colored lines indicate data from individual participants. Time courses are provided in Figure S2. Acronyms: BL, baseline; SD, standard deviation; WMZ, wake maintenance zone.

To explore further what drives the different trajectories observed in the EEG data across wake, and in particular the changes in the WMZ, we analyzed pupillometry data from the Fixation and Oddball conditions. In both, mean pupil diameter after an initial decrease remained largely constant during the extended wake period (Figure 7A), with a specific increase during the two WMZ timepoints (Fixation t_(15)_ = 4.65; Oddball t_(12)_ = 2.17, p = 0.051). There was also a significant drop in pupil diameter from S1 to S8 during Fixation (t_(16)_ = −3.78, p = 0.002) but not Oddball (t_(12)_ = −1.39, p = 0.189). Interestingly, the two baseline recordings done in the evening an hour before bedtime (BL Pre, Pre) showed larger diameters during Oddball than during Fixation (BL Pre: t_(12)_ = 2.18, p = 0.050, g = 0.62; Pre: t_(16)_ = 3.22, p = 0.005, g = 0.63), but not during the same recording of extended wake (S7 t_(14)_ = −0.77, p = 0.453, g = −0.16). This indicates an interaction, at least within the WMZ, between recording condition, pupil diameter, and prior sleep restriction.

Standard deviations of pupil diameter were also assessed (Figure 7B). Across sleep, there was a large significant decrease in standard deviations for both conditions (Fixation t_(13)_ = −3.76; Oddball t_(12)_ = −3.66). During extended wake, standard deviations increased to maximum values in the afternoon (S4, 15:00). Standard deviations then tended to decrease during the WMZ, although the effect was only significant in the Oddball (t_(12)_ = −2.58, p = 0.024) and not Fixation (t_(15)_ = −1.69, p = 0.111). Across wake there was a significant increase in standard deviations from S1 to S8 (Fixation t_(16)_ = 3.52; Oddball t_(12)_ = 4.74), although S8 Oddball values returned to those of S3-S5 after the WMZ, whereas S8 Fixation remained lower.

**Figure 8:**
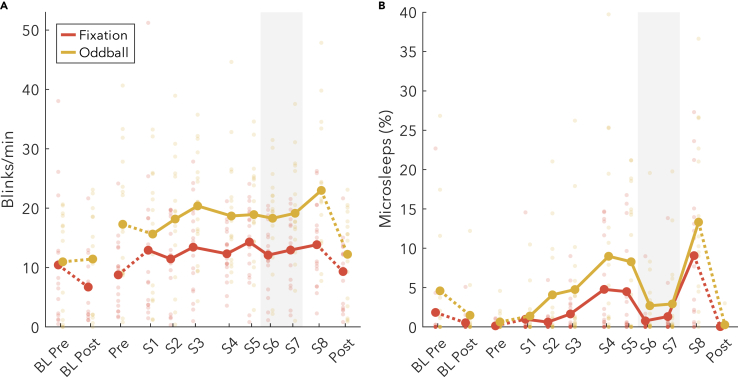
Eye-closures (A) Number of blinks per minute. A blink is defined as any eye-closure less than 1 s long. (B) Percent of recording with ocular microsleeps (eyes closed longer than 1 s). N.B. here raw values are provided, although the statistics described in the text are with z-scored values. Thick lines indicate group averages for each condition across sessions (x axis), with color indicating condition. Solid lines connect sessions during the same-day extended wake period, and dashed lines indicate changes across sleep. S1–S8 are spaced out relative to the time they occurred within the 24 h wake period (Figure 1B). Dots reflect individual participants’ datapoints. The shaded gray area indicates the WMZ. Acronyms: BL, baseline; WMZ, wake maintenance zone.

Finally, we investigated the pupil response to target tones during the auditory Oddball condition (Figures 7C and S2). Unfortunately, there was substantial data loss due to increased eye closure with extended wake (Figure 8B) combined with equipment malfunctions during measurements, so power for this analysis is reduced. Pupil response to oddball tones was quantified as the area under the curve between the pupil response to targets relative to standard tones, from 0.5 to 2 s after tone onset. There was no change in response to targets from evening to morning around a baseline night of sleep (t_(9)_ = 0.26), nor was there a significant change from beginning to end of the extended wake period, although there was on average a decrease in pupil oddball response (t_(11)_ = −1.58). There was trending effect of the WMZ (t_(9)_ = 2.08, p = 0.067). However, as can be seen from Figure 7C, this was almost entirely due to a drop in the oddball response for S8 relative to previous sessions (e.g. S5 vs. S8: t_(10)_ = 2.77, p = 0.020, g = −1.07). Furthermore, there was never a return to baseline values of pupil oddball response following recovery sleep (pre vs. post: t_(13)_ = 2.43, p = 0.030, g = −0.62), complicating any potential interpretation.

**Figure:**
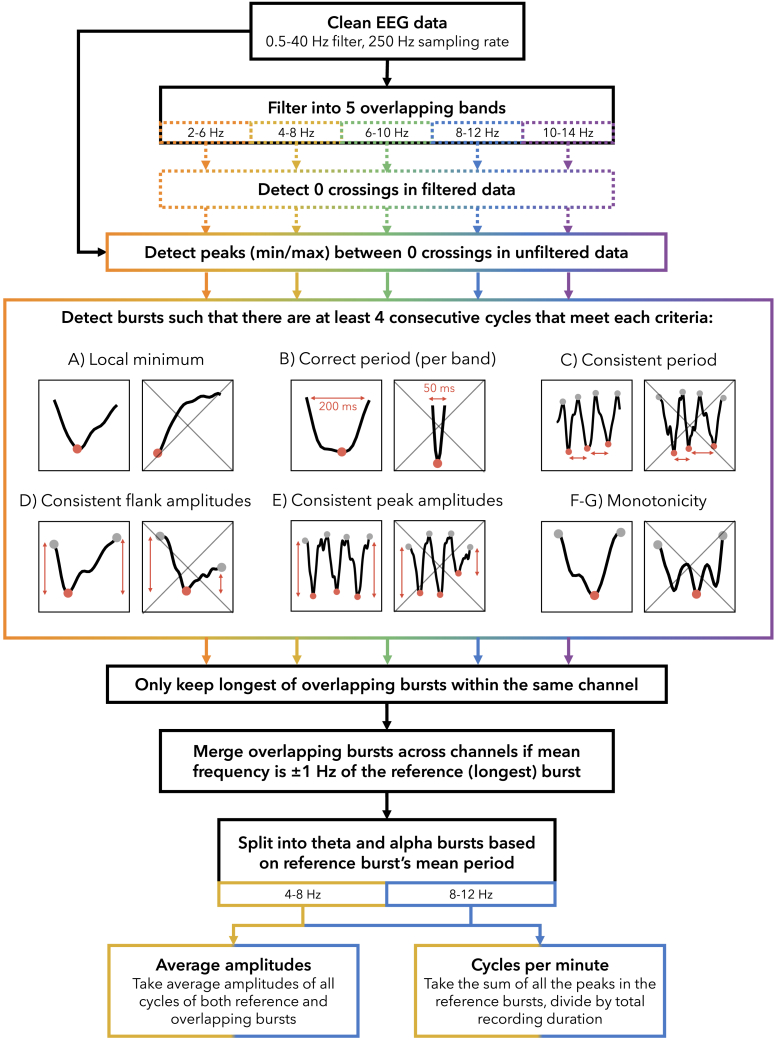
Burst detection algorithm Solid outline indicates processes conducted on “unfiltered” data (only filtered between 0.5 and 40 Hz), dotted lines indicate processes conducted on filtered data (in 4 Hz bands). Colors indicate data processed separately for each band, black indicates processes done on pooled/undifferentiated data. A–G are examples of cycles that do or do not meet the criteria (crossed out). A and B depict half-cycles, from zero-crossing to zero-crossing. D and F/G indicate a whole cycle, from positive peak to positive peak. C and E indicate 3 consecutive cycles. In cycle-examples, red circles indicate the negative peaks, and gray circles positive peaks.

### Ocular microsleeps are sensitive to the WMZ, blink rates are not

In addition to actual pupil diameters, the same eye-tracking cameras were sensitive to ocular behavior such as blinking and microsleeps, both of which increase with sleepiness^38^^,^^39^ and can potentially affect measures of pupillometry. We therefore wished to determine whether the changes in pupillometry could be explained by ocular behavior, based on whether they followed similar trajectories across time. We measured all eye closures and split them into blinks when less than 1 s,^40^^,^^41^ and as ocular microsleeps when longer than 1 s^42^ We specify “ocular” to distinguish from microsleeps properly classified with the EEG,^42^ and we label them as “microsleeps” rather than merely eye closures because prolonged eye closures during sleep deprivation are predictive of behavioral lapses in auditory tasks.^43^

Blink rates (Figure 8A) gradually increased across wake in the Oddball (t_(13)_ = 4.37) but not Fixation condition (t_(16)_ = 0.62), and there was no change during the WMZ (Fixation t_(15)_ = −1.25; Oddball t_(15)_ = −0.21). Therefore, blinking could not explain changes in pupillometry. At the same time, the amount of ocular microsleeps was extremely sensitive to the WMZ (Fixation t_(15)_ = −4.85; Oddball t_(15)_ = −6.28), returning almost entirely to baseline levels. However, across extended wake, ocular microsleeps otherwise increased linearly, starting from near zero and reaching 10–15% of the recordings by S8 (Fixation t_(16)_ = 4.81; Oddball t_(13)_ = 4.18). Like with blinking, this trajectory was not reflected in the various measures of pupillometry, and therefore cannot explain the results.

## Discussion

The two-process model of sleep has been the backbone of sleep research for the past 40 years, and has withstood the test of time.^44^ Not only is sleep homeostasis uncontroversial, it is now used to prove whether a given organism sleeps at all.^45^^,^^46^ The synaptic homeostasis hypothesis has been more contentious, especially in the details of the underlying mechanisms behind synaptic “strengthening,”^47^^,^^48^^,^^49^ however, the general idea is compelling: that synaptic changes during the day requires sleep to restore synaptic balance. Both of these models were based primarily on observations during sleep, and while they made testable predictions of what should occur during wake, measurements of EEG power did not conform to expectations. The two-process model of sleep postulated that sleep need accumulates along an increasing saturating exponential function with time awake (Figure 1A), and the synaptic homeostasis hypothesis proposed that this process is driven by increasing synaptic strength, which results in increased neuronal synchrony.^8^^,^^12^ By separately analyzing oscillation amplitudes and quantities, we were able to validate these predictions.

Our results reveal that indeed both theta and alpha amplitudes follow an increasing saturating exponential trajectory across extended wake, following the predicted trajectory of sleep homeostasis (Figures 5A and 5B vs. Figure 1A). Furthermore, both theta and alpha amplitudes returned to baseline levels following recovery sleep, with trending decreases for theta and highly significant decreases for alpha around baseline sleep. These results, therefore match the predictions of the two-process model and the synaptic homeostasis hypothesis. It is still possible that synaptic strength does not drive this increase in amplitudes, but at the very least, changes in wake oscillation amplitudes correspond to the changes observed in slow wave activity.^8^^,^^10^^,^^11^

In particular, the fact that the number of alpha oscillations decreased at the same time as amplitudes increased, and the former was widespread and the latter was localized (Figure 6D), clearly indicates a dissociation between these homeostatic changes in amplitude from whatever process causes oscillations to occur in the first place. More subtly, while both theta amplitudes and quantities increased during extended wake, the trajectories were different, suggesting again distinct mechanisms. In both cases, spectral power was insufficient to capture these trends with time, as it cannot differentiate between simultaneous and comparably large changes in amplitudes and quantities.

These results invite re-analysis of many previous findings showing differences in power between conditions and populations. Efforts are already underway to distinguish the effects of periodic and aperiodic components of the EEG spectrum.^31^ Therefore, in cases in which the effect is largely periodic, further investigation should determine whether it is the amplitude or quantities of oscillations that change; this can then provide more interpretable results. In the context of understanding synaptic plasticity for example, it would be beneficial to re-analyze data from the studies of Hung et al.^50^ and Bernardi et al.^51^ These two landmark studies found that theta power in resting state EEG increased locally depending on prior daytime activity. Re-analysis of these datasets may reveal whether this effect was specific to oscillation amplitudes, even in the alpha band, which would further support the interpretation that such local effects are driven by spatial differences in synaptic strengthening.

### Sensitivity of different conditions to sleep homeostasis

Beyond demonstrating whether the standard eyes-open resting EEG would be sensitive to sleep and synaptic homeostasis, we were interested in establishing whether this generalized to other conditions. We indeed found that the EEG of the Oddball followed the same trajectories as Fixation, but the Standing condition did not. Theta Standing amplitudes did not increase past S3 during the extended wake period, and alpha Standing amplitudes neither decreased following sleep, nor did they increase during extended wake past S2. Given the overall larger amplitudes in this condition, this may be at least partially due to a ceiling effect. At the same time, given the eyes-closed condition, it is also possible that participants were closer to “true sleep” during S8 Standing. This is supported by the complete change in spectrogram in the Standing Back channels (Figure S1). Altogether, our results indicate that oscillation amplitudes tend to reflect sleep homeostasis; however, this is not the only factor contributing to these amplitudes. In practice, this means that different recording conditions will be more or less sensitive to changes in sleep homeostasis.

### Cortical desynchronization during the WMZ

What did not match our predictions at all were oscillatory amplitudes during the WMZ. In both Fixation and Oddball, theta and alpha oscillation amplitudes decreased during the WMZ, and then returned to their previous trajectories during S8. The effect sizes of the WMZ were actually larger for amplitudes compared to the number of oscillations (Table 1). The fact that oscillation amplitudes decreased implies that whatever mechanism drives the WMZ, it results in overall desynchronized cortical activity. Supporting this finding of cortical desynchrony, Ly et al. found that transcranial-magnetic stimulation (TMS) evoked potentials, reflecting cortical excitability, had lower amplitudes during the WMZ compared to earlier in the day.^52^ Except for such circadian changes, TMS evoked potentials across the day reflect sleep homeostasis, increasing with time awake and decreasing following sleep.^53^ However, more generally, increased cortical synchronization reflects decreasing levels of consciousness, with deeper sleep stages and comatose states producing the largest evoked potentials.^54^^,^^55^^,^^56^ Therefore it is possible that the WMZ may even reflect a qualitatively distinct state of alertness.

In practice, this means that oscillation amplitudes can usually be used as a marker for homeostatic sleep pressure, except during the hours before habitual sleep. We do not consider this dip in amplitudes during the WMZ (and the difference in Standing oscillation amplitudes described previously) to indicate that the two-process model and synaptic homeostasis hypothesis are inaccurate, just that the amplitudes of oscillations are no longer accurately reflecting the wake-related changes in neuronal connectivity and sleep homeostasis.

### Timing and duration of the WMZ

It is noteworthy how the WMZ briefly interrupts both the linear increase in theta quantities and the saturating exponentials of theta and alpha amplitudes. This highlights how the WMZ is limited in time, unlike the gradual circadian component of the two-process model (Figure 1A). The model was established based on measures of alertness, core body temperature, and melatonin concentration, all of which changed approximately sinusoidally across 24 h.^21^ While none of our outcome measures were paralleling these sinusoidal fluctuations, almost all of them were clearly affected by the brief WMZ. We therefore suspect that a specific pathway is responsible for the WMZ, distinct from melatonin concentration and core body temperature, although still synchronized to the suprachiasmatic nucleus (SCN), the brain’s timekeeper.^57^

The timing of the WMZ in our study diverges slightly from some previous studies which find the WMZ to occur 3–6 h before bedtime,^23^^,^^27^^,^^29^ but is in agreement with others that find the WMZ 1–4 h before bedtime.^24^^,^^25^ Studies like ours with later WMZ times were conducted under normal office lighting conditions (∼150 lux, or no manipulation reported), whereas studies with earlier WMZ times were recorded under dim lighting conditions (< 10 lux). As demonstrated by Gooley et al.,^58^ brighter daytime light shifts melatonin onset to around 2 h later, likely explaining the difference in results. This therefore means that whatever light-induced mechanism delays melatonin onset, it also delays the WMZ onset.

### Neural pathways of the WMZ

Given that both the mean and standard deviation of pupil diameters were also strongly affected by the WMZ (Figure 7), it is likely that the AAS is involved. The AAS includes the LC, the ventral tegmental area and substantia nigra, the dorsal and median raphe nuclei, and the basal forebrain.^36^ All these areas have been linked to changes in pupil diameter,^36^^,^^37^^,^^59^ and have widespread connections to the rest of the cortex. The LC in particular has been linked to transitory pupil responses such as the increases observed during oddball tasks.^33^^,^^34^^,^^35^ Since we do not find an increase in pupil responses to oddball target tones in the WMZ (Figure 7C), this may suggest that of all the AAS, the LC is actually not involved in the WMZ. Alternatively, this may merely indicate that pupil oddball responses are not a reliable indicator of LC activity across time. Unfortunately, due to reduced power, these results are suggestive at best, and furthermore the link between LC and pupil diameters has not been unambiguously established. Hopefully future studies will be able to determine which nuclei are involved in the WMZ.

### The role of the WMZ in humans

The WMZ has, to our knowledge, only been investigated in humans. It is possible the WMZ may even be human-specific, but this can be difficult to validate because it bears a close resemblance to crepuscular behavior observed in some animal species, especially rodents.^60^^,^^61^ Crepuscular rhythms manifest as increases in activity at dawn and dusk, usually accompanied by corresponding increases in core body temperature.^62^^,^^63^ By contrast, the WMZ in humans occurs in the absence of either increases in activity^64^^,^^65^ or core body temperature,^25^ suggesting differences in underlying physiological pathways, as well as different functions.

The fact that the WMZ primarily affects sleepiness and sleep latencies suggests that the main function of the WMZ is to resist sleep rather than promote activity. This may be a human-specific adaptation because we have long consolidated wake and sleep, unlike most other species who have more fragmented and polyphasic sleep.^66^^,^^67^ By ensuring that individuals do not initiate sleep too early, the WMZ largely guarantees that the subsequent 8 h of sleep are *completed* within the correct circadian window, thus maintaining continued synchronization with the overall circadian rhythm and environmental light-dark cycles. During normal wake, the WMZ may not be apparent or even necessary, however, when homeostatic sleep pressure is unusually high (for example from insufficient sleep the night before), such a mechanism would be critical to maintain wakefulness until the onset of the correct sleep window. In polyphasic-sleep species such as mice and rats, the timing of sleep onset for any given sleep episode is less critical. An alternative hypothesis is that the WMZ is a vestige of primate nest-building, which also occurs in the evening. However, such an activity takes around 7 min^68^ so this does not explain why the WMZ would last several hours.

The WMZ needs to be investigated more. As speculated by Strogatz et al.,^22^ such a mechanism may be behind sleep disorders such as insomnia; if the WMZ never “shuts off,” this will result in substantially delayed sleep onset; if it is not present at all, this could result in circadian desynchrony. Taking this one step further, control over the WMZ could improve general wellbeing; being able to selectively shut it off could help with jetlag. Alternatively, activating the WMZ during night shifts could improve performance in critical industries, such as emergency medicine or airline pilots. Our 4/24 extended wake paradigm, measuring both EEG and pupillometry, consists of a comparatively easier and equally effective approach to investigating the WMZ relative to the standard > 40 h sleep deprivation, and may therefore be better suited for large-scale studies also involving patients.

### Limitations of the study

While we are satisfied with many of the design choices for this experiment, there is room for improvement. Given the unexpected importance of the WMZ, we would have benefitted from a traditional circadian marker such as melatonin concentration, which would have allowed more precise synchronization across participants to circadian phase. Additionally, more frequent recordings (e.g. every hour) would have provided a better temporal resolution to delineate the start and end of the WMZ for each participant. Regarding the wake recordings, given that the Oddball condition produced the largest effect sizes, its possible more controlled tasks will provide better results than Fixation. Regarding the analyses, we implemented a relatively basic cycle-by-cycle burst detection algorithm, and there is ample room for improvement following more systematic development of this approach.

Regarding the interpretation of the EEG results, it is important that they are replicated with datasets using much longer bouts of sleep deprivation, spanning more than 24 h and with more than a single recording after the WMZ; it is possible that slower circadian changes are still present in oscillation amplitudes, which cannot be disentangled within a single period. Likewise, data exists using the forced desynchrony protocol^18^ which can properly dissociate circadian from homeostatic changes, as well as a constant routine protocol to control for homeostatic pressure.^69^ It would be important to see to what further extent amplitudes and number of bursts differently reflect circadian and homeostatic changes. Regarding the pupillometry results, it is important that they are replicated with larger sample sizes, and possibly comparing circadian changes with and without sleep restriction, as our results suggest an unexpected interaction.

### Conclusions

In summary, we found that wake EEG oscillations reflect the increase in neuronal connectivity that builds up with time awake, through increased amplitudes, which change independently across time from oscillation quantities. We demonstrated that both theta and alpha amplitudes follow the same increasing saturating trajectories of sleep homeostasis previously identified with slow waves during sleep. However, the WMZ proved to be such a potent contributor to wakefulness as to temporarily counteract these effects, impacting both the amplitudes and number of occurrences of oscillations. In addition to the EEG, we have identified mean pupil diameter as specifically sensitive to this window. This specificity strongly suggests that the WMZ is caused by a wakefulness driver distinct from the gradual sinusoidal 24 h circadian fluctuations in alertness. Finally, we speculate that the AAS may be crucially involved, and that the WMZ may be a human-specific mechanism to resist sleep under unusually elevated levels of homeostatic sleep pressure.

## STAR★Methods

### Key resources table

**Table:** 

REAGENT or RESOURCE	SOURCE	IDENTIFIER
**Deposited data**		

All preprocessed data, raw pupil sizes, clean Fixation EEG, detected bursts.	Mendeley Data	https://doi.org/10.17632/72k5vcwk7n.1

**Software and algorithms**		

Matcycle, custom MATLAB code for cycle-by-cycle detection of EEG bursts.	Zenodo	https://zenodo.org/badge/latestdoi/507885968
Custom analysis scripts	Zenodo	https://zenodo.org/badge/latestdoi/508216437
Preprocessing scripts	Zenodo	https://zenodo.org/badge/latestdoi/353021931

### Resource availability

#### Lead contact

Further information and requests for data should be directed to and will be fulfilled by the lead contact, Sophia Snipes (snipes.sophia@gmail.com).

#### Materials availability

There are no newly generated materials to report.

### Experimental model and study participant details

18 participants completed the experiment. University student applicants were screened for good health, good sleep quality, and at least some sleep deprivation vulnerability. 19 participants were recruited, and one participant dropped out midway. Of the 18 participants who completed the experiment, 9 were female and 3 were left-handed, all of European ancestry. Between-sex comparisons were not considered due to the small sample size and consequent low power. Participants were required to be between the ages of 18 and 26 in order to reduce interindividual age-effects of sleep homeostasis; consequently, mean age (± standard deviation) was 23 ± 1 years old. This limits generalizability, but the outcomes for specific populations can be anticipated from homeostatic changes in slow wave activity. All participants self-reported no hearing impairments. Data collection and interaction with participants was conducted according to Swiss law (Ordinance on Human Research with the Exception of Clinical Trials) and the principles of the Declaration of Helsinki, with Zurich cantonal ethics approval BASEC-Nr. 2019-01193. Participants signed informed consent before the study.

### Method details

#### Experiment design

Participants conducted a 4/24 extended wake paradigm, depicted in Figure 1. This involved habituation to a regular sleep-wake schedule prior to the experiment (minimum 4 nights), with bedtimes and wakeup times selected to match the participant’s preferred window and daily schedule. During the experiment, participants went to bed at their habitual bedtime, and were woken up 4 hours later. They were then kept awake for 24 h, followed by a recovery night. In addition to the extended wake bout, participants conducted a baseline night in which they slept during their habitual sleep window. The baseline was conducted before the extended wake bout in all but four participants.

The experiment schedule is in Figure 1B. Resting wake EEG recordings were measured before and after each night of sleep, and an additional 6 times during the 24 h wake period, for a total of 12 recordings. Prior to each recording S2-S7, participants were seated in the same position, watching 2 TV episodes around 40 minutes each, from a series of their choice. After each rest recording, participants were free to move around, and were provided with a home-cooked meal which they had selected from a list of vegetarian options (each meal during each break was the same). 6 of these breaks were included, each around 40 minutes (adjusting for delays in the schedule).

During the rest recordings, participants were seated in a comfortable armchair with a footrest (IKEA strandmon) in a well-lit room (∼150 lux at eye level) and had to maintain fixation on a 20 cm red cross placed ∼3 m from their head, ∼30 cm below eye-level. The timing of the three rest recording conditions is depicted in Figure 1C. Each session began with a Fixation period, in which their only instructions were to maintain fixation on the cross and stay awake. This was immediately followed by an active auditory Oddball. As the focus was on pupillometry for this task, the stimuli were tones rather than visual inputs in order to avoid any spurious changes in light. Two types of tone were presented: standards (160 tones), and targets (40 tones). Participants had to press a button whenever a target tone occurred, while maintaining fixation and staying awake. Each tone lasted 60 ms, and for each participant the tone was either 660 Hz or 440 Hz for the targets, and vice versa for the standard tones. The interstimulus interval ranged randomly from 1.8 to 2.4 s, with a minimum of 3 standard tones between targets. After the Oddball, participants were provided a questionnaire to fill out, including the Karolinska Sleepiness Scale (KSS, Figure 2).^71^ Finally, participants stood up from the chair and moved to lean against the wall and had a Standing period with eyes closed. The purpose of this condition was to have a long recording with eyes-closed, the most typical condition for alpha activity,^72^ without participants falling asleep. Caldwell et al.^73^ previously found that there was no effect on alpha activity when comparing seated to standing recordings across sleep deprivation during eyes closed.

Participants maintained a regular sleep-wake schedule the week prior to each experiment bout, and the timing of the experimental nights was adapted to each individual’s preferred circadian time. Therefore the 24 h circadian rhythm could be inferred from previous studies.^21^^,^^25^^,^^74^ Changes synchronized to melatonin would be high during night recordings (S1, S2, S8), and low during day recordings. Vice versa, circadian changes synchronized to core body temperature would peak in the middle of the day (S4, S5), and be low in the middle of the night (S1, S8). Because the focus was on homeostatic changes, these comparisons were not statistically analyzed, but can be inferred from the outcome measures’ trajectories.

#### EEG preprocessing & power analysis

EEG data was recorded at 1000 Hz, with 129 electrodes including the Cz reference, using EGI HydroCel Geodesic Sensor nets. Four electrodes were external to the net, and were positioned on the mastoids and under the chin for sleep scoring (sleep architecture is available in Snipes et al.^70^). Two electrodes (126, 127) were located on the cheeks and excluded, leaving 123 channels for EEG data analysis after re-referencing to the average. Preprocessing and data analyses were done using EEGLAB^75^ and custom MATLAB scripts. Data was downsampled to 250 Hz and filtered between 0.5-40 Hz. Major artifacts were identified visually, and physiological artifacts (eye movements, heartbeat, muscle activity) were removed with independent component analysis (ICA). The process is depicted in detail in Snipes et al.^70^ Supplementary Figure 5-1.

Power was calculated as power spectral density using Welch’s method with 8 s windows, Hanning tapered, 75% overlap. Power values for each participant and each frequency were z-scored pooling across sessions, conditions, and channels. Z-scored values were then averaged across all channels excluding the outer-edge electrodes (Figure 3). When plotting spectrograms, a 1 Hz lowess filter was used to smooth the signal (Figure S1).

#### EEG burst detection

There were two main reasons for analyzing EEG oscillations as bursts using cycle-by-cycle analysis. First, when visually inspecting the EEG during extended wake, the most prominent features are in fact bursts rather than single isolated events, as can be seen in the example of Figure 4. Second, any method trying to investigate independent changes in oscillation amplitude and quantity must ensure that the detection method does not rely on either. Previous studies investigating plasticity-dependent effects and local sleep in wake identified waves based on either fixed or relative voltage thresholds.^50^^,^^51^^,^^76^^,^^77^^,^^78^ The problem with fixed thresholds when trying to answer our research question is that when oscillations increase in amplitude without changing in quantity, they will still *appear* to increase in quantity since more waves are now above-threshold. The additional problem with relative thresholds, such as taking the top N% of all waves recorded for a given participant, is that it constrains the number of detected oscillations independently of how prevalent they are in the signal. Therefore, if a given participant has no oscillations, this method will identify false positives from the 1/f aperiodic background activity. Vice-versa, if a participant has a recording completely dominated by oscillations (such as with eyes-closed), this method will miss most of them. The problem of dependency between quantity and amplitude, as well as the problem of both over- and underestimating oscillations, persists for all oscillation-detection methods that require amplitude cutoffs, including wavelets and Hilbert.^30^ Cycle-by-cycle analysis avoids this by relying entirely on the shape and regularity of the signal; the tradeoff is that it is not suited to detecting single isolated waves such as local sleep events.^79^

For our analysis, burst detection was conducted with custom MATLAB scripts adapted from Cole and Voytek’s Python *bycycle* package.^32^ The pipeline described below is also provided schematically in below figure. First, clean EEG data was filtered into narrow overlapping bands (2–6, 4–8, 6–10, 8–12, 10–14 Hz) using a minimum order high-pass then low-pass equiripple FIR filter (stopband frequency = passfrequency ± 1 Hz, passband ripple 0.04 dB, stopband attenuation 40 dB). Zero-crossings were identified in the narrow-band filtered data. Then between descending zero-crossings and rising zero-crossings, negative peaks were identified as the minimum value in the “unfiltered” data (minimally filtered during pre-processing between 0.5 and 40 Hz). Positive peaks were also identified as the maximum values in the unfiltered data between rising and descending zero-crossings, and these were used as the start and end of each cycle around the negative peak.

Once all the peaks were identified, 4 consecutive cycles had to meet the following properties in order to qualify as an oscillation burst: A) the cycle’s negative peak had to correspond to a local minimum; B) the mean distance to the neighboring peaks had to be within the range of the period of the filter (e.g. between 0.1 – 0.17 s when filtering between 6-10 Hz); C) the minimum ratio between the distance in time of the current peak to its neighbors had to be above 0.6 (i.e. similar consecutive periods); D) the rise amplitude, measured as the voltage difference between the prior positive peak to current negative peak, and decay amplitude of the cycle had to have a ratio of at least 0.5 (i.e. one flank was not less than half the amplitude of the other); E) the minimum ratio between the cycle amplitude (negative peak to positive peak voltage, averaging both neighboring positive peaks) and neighboring cycles had to be more than 0.6 (i.e. similar consecutive amplitudes); F) the proportion of timepoints decreasing in amplitude between previous positive peak and current negative peak, and timepoints increasing in amplitude between current peak and following positive peak, had to be above 0.6 (i.e. how much *time* during the cycle the signal went in the wrong direction); and G) the proportion of the voltage increasing from positive to negative peak, and decreasing from negative to positive peak, had to be above 0.6 (i.e. how much *amplitude* went in the wrong direction). The criteria B, E, and F are from Cole and Voytek, whereas A, C, D, and G are our additional optimizations. The parameters and burst-detection criteria were chosen through trial-and-error on an independent subset of data recorded during this experiment (the Game and PVT conditions of the SD task block reported in Snipes et al.^70^). The procedure involved iteratively adjusting thresholds and introducing cycle exclusion criteria until the theta and alpha burst detection was largely consistent with visual inspection.

Bursts were detected for all frequency bands, using both the EEG signal and the inverse of the EEG signal (because for mu-shaped rhythms, the sharper peaks resulted in better burst detection). Within each channel, overlapping bursts were compared, and the largest was retained intact. Smaller partially overlapping bursts were cut, and if the non-overlapping segment still retained 4 cycles, it was considered a new burst. Then, bursts were aggregated across channels based on temporal overlap (at least 50%) and if the mean frequency was within 1 Hz for the overlapping cycles.

Burst frequencies were defined as the reciprocal of the mean distances between negative peaks. Burst amplitudes were calculated by first averaging the rise and decay amplitudes of each cycle, then the average of these across all cycles in the aggregated bursts in different channels, then averaging the amplitude of all bursts within the band of interest. Burst quantities were calculated as the sum of all the cycles in the reference burst (the longest of all the overlapping bursts), divided by the duration of the recording, resulting in cycles per minute. This was chosen instead of the total number of bursts per minute because in extreme cases, bursts could become so long that their quantity decreased, and this was no longer representative of their occurrence in the data.

Figures S3A–S3B plots the distribution of the number of bursts by frequency for two example participants. Cycle-by-cycle analysis allowed clear differentiation between clusters of bursts by frequency. However, many individuals did not have two (theta/alpha), but in fact three or more clusters, and these distributions changed with time awake (best example: Figure S3A, Fixation). At the same time, other participants showed more classical bimodal distributions (Figure S3B). Ideally, we would have used individualized bands to delineate theta and alpha, however these shifting distributions complicate such an approach. Therefore, we limited ourselves to group average results using traditional frequency bands. Furthermore, rather than quantifying the occurrence of a given oscillation by the number of bursts as shown in Figures S3A–S3C, we used instead the number of cycles per minute (Figure S3G), as this also captures changes in burst duration (Figure S3E).

#### Eye tracking & pupillometry

Eye tracking was done with Pupil Core “glasses” (Pupil Labs, Berlin, Germany). These were eyeglass frames with two rear-facing infra-red cameras. Pupil diameter was estimated from the video recorded with a sampling rate of on average 120 Hz. Data was exported using Pupil Player. All analyses were then conducted with a sampling rate of 50 Hz. During measurements, the eye tracker failed multiple times, resulting in substantial data loss. Sleep loss further resulted in noisier data (more eye-closure, less fixed gaze, half-closed eyelids).

The eye tracking variables blink rate and ocular microsleeps were measured using the confidence values of the pupil diameter estimates (from 0 to 1): when model confidence fell below 0.5, this was considered an eye-closure. This approach was chosen based on our observation of the video relative to the model confidence. Consecutive timepoints with confidence values over 0.5 that lasted less than 50 ms were still considered eyes-closed, and consecutive timepoints under 0.5 and less than 50 ms long were considered eyes open. The cutoff to split blinks and microsleeps was based on previous research identifying microsleeps as short as 1 s.^42^

2D pupil diameter was estimated from the eye videos offline with Pupil Player, measured in pixels. Between the recordings, the eye tracking glasses were removed and readjusted. Therefore, in order to compare mean pupil diameter across sessions, the diameters in pixels had to be re-scaled. For every video, a frame was selected (192 × 192 pixels, 4.5 × 4.5 cm), and the eye’s iris diameter was measured in centimeters (when viewed at an angle, a disk becomes an ellipse, and the largest diameter of the ellipse is the diameter of the disk). By using the human mean iris diameter (12 mm), a conversion factor was calculated between pixels and millimeters, and this was applied to all 2D pupil diameter measurements:

$$pupil\;\left(mm\right)=\frac{pupil\;\left(px\right)\times video\;width\;\left(cm\right)\times standard\;iris\;\left(mm\right)}{iris\;\left(cm\right)\times video\;width\;\left(px\right)}$$

While this does not preserve individual differences in eye-size, it is sufficient for comparing across-session changes in diameter within participants (reasonably assuming irises do not change in size with sleep pressure). Furthermore, it allows the exclusion of unphysiological outliers of diameter estimates.

Pupil preprocessing was done with the PhysioData toolbox.^80^ Finally, removed datapoints less than 0.5 s were linearly interpolated, and then isolated chunks of datapoints less than 0.5 s were removed. Only data from one eye was used for each participant. The eye was chosen based on which had the most data after preprocessing.

To measure pupillary response to deviant tones during the Oddball, pupil diameters were epoched between −0.5 and 2 s relative to tone onset. All 40 targets were used, with 40 standards taken from the trial just prior to each target. Trials with less than 2/3 of clean timepoints were excluded. Recordings with less than 15 trials for either targets or standards were excluded. Furthermore, if any *timepoint* for a given tone type was derived by averaging fewer than 10 trials, this session was also excluded.

For each trial, the pupil response to tones was first baseline corrected (the mean between −0.5-0 s was subtracted from all datapoints in the trial), then all trials were averaged for each recording, split by target and standard tones. Participants with fewer than 6 recordings out of the 12 were excluded. Finally, average pupil responses for all timepoints, both targets and standards, and all sessions were z-scored within each participant. Pupillary response was calculated as the area under the curve between 0.5 and 2 s between target and standard.

### Quantification and statistical analysis

To quantify the effects of extended wake, sleep, and the WMZ, for each outcome measure we conducted the same three paired t-tests. For wake-dependent changes, we compared values from the start and end of the 24 h extended wake period, S1 and S8. These were within 2 h of the same circadian phase, therefore any differences should largely be due to sleep homeostasis. To quantify sleep-dependent changes, we compared values from the wake recordings before and after the baseline night, BL Pre and BL Post. Unlike for wake changes, these were conducted at different circadian times and the difference in sleep homeostatic pressure was lower, however these are typical recordings during sleep studies.

To statistically quantify any deviation during the WMZ from the underlying trajectory of a given outcome measure, we linearly interpolated values from S5 (17:30) to S8 (2:40) for timepoint 21:30 and compared it to the average of S6 (20:00) and S7 (23:00). The timepoints of the WMZ were determined based on the converging results of subjective sleepiness (Figure 2) and theta power (Figure 3A), both of which showed a decrease in an otherwise monotonic increase during recordings S6 and S7, corresponding to 1–4 h before habitual bedtime.

All statistics were paired t-tests, such that p < 0.05 was considered statistically significant. All t-tests were conducted on z-scored values (pooling sessions and conditions for each participant) to better account for interindividual differences, provide more normally distributed datapoints, and more fair comparison of effect sizes across outcome measures. Due to occasional data loss for different outcome measures, the degrees of freedom are always provided, from which the sample size can be inferred (N = DF + 1).

Tests were selected a-priori for BL Pre vs BL Post to quantify overnight changes, and S1 vs S8 to quantify wake changes. To quantify the WMZ, a single value based on the average of the two recordings systematically showing effects (S6 and S7) were used. These were compared to an “expected” value based on S5 and S8, linearly interpolated. While previous studies quantified the effect by comparing WMZ values with measurements just prior, we considered this an under-estimate of the effect, as it doesn’t take into account the overall trajectory of the data, i.e. what values those timepoints would have had without the presence of the WMZ. However, our method can also overestimate the WMZ, if either S5 or S8 deviated substantially from the rest of the recordings. Therefore, results were interpreted in the context of the trajectories observed in the figures.

Hedge’s g effect sizes were reported for each test in Table 1, calculated with the Measures of Effect Size Toolbox.^81^ Effect sizes are typically evaluated with Cohen’s rule-of-thumb such that g values < 0.2 are “small”, around 0.5 “medium”, and > 0.8 “large”.^82^ To determine what effect sizes we had enough power for, we conducted a post-hoc statistical power analysis using standard values of α = .05 and 1-β = .8. For an N = 18, we had power for effect sizes of Hedge’s g ≥ 0.68, and N = 10 had power for Hedge’s g ≥ 0.95. While this is generally a limitation, both sleep deprivation effects and WMZ effects tend to be quite large.^23^

No correction for multiple comparisons was done for these statistical tests as the majority were exploratory (e.g., Oddball/Standing conditions, pupil measures) or confirmatory (e.g., if amplitudes increase across wake, they should also decrease after sleep). Furthermore, there was a mixture of dependent and independent comparisons (e.g., power = amplitudes + quantities). All these t-tests were calculated in order to quantify the changes across outcome measures and thus compare effect sizes and relative robustness. The main hypothesis of whether both theta and alpha oscillation amplitudes increased with extended wake was a-priori selected for the Fixation condition. False-discovery rate correction^83^ was however conducted for the 123 t-tests in each of the topographies of Figures 6C and 6D.

Throughout the text, the changes in average oscillation amplitude across extended wake are provided as average percent change from S1, with interquartile range (25% and 75% of the individuals) provided in brackets. This was used instead of standard deviation to better represent potentially skewed distributions. Likewise, the change in quantities of oscillations are described in the text as percentage of the entire recording, with corresponding interquartile ranges.

### Additional resources

Original cycle-by-cycle implementation: https://github.com/bycycle-tools/bycycle.

The plotting graphics: https://github.com/snipeso/chART.

The two-process model in Figure 1A: https://github.com/HuberSleepLab/2processmodel.

The pupil preprocessing toolbox: https://github.com/ElioS-S/pupil-size.

## Data Availability

• The raw data reported in this study cannot be deposited in a public repository because it is too large. To request a copy, contact Sophia Snipes (snipes.sophia@gmail.com) or Reto Huber (reto.huber@kispi.uzh.ch). Summary values used for the statistics in Table 1, as well as detected bursts, preprocessed EEG of the Fixation condition, and pupillometry have been deposited at Mendeley Data and are publicly available as of the date of publication. The DOI is listed in the key resources table. • All original code has been deposited at Zenodo and is publicly available as of the date of publication. DOIs are listed in the key resources table. • Any additional information required to reanalyze the data reported in this paper is available from the lead contact upon request.

## References

[1] Vyazovskiy V.V., Harris K.D. (2013). Sleep and the single neuron: the role of global slow oscillations in individual cell rest. Nat. Rev. Neurosci..

[2] Hauglund N.L., Pavan C., Nedergaard M. (2020). Cleaning the sleeping brain – the potential restorative function of the glymphatic system. Current Opinion in Physiology.

[3] Xie L., Kang H., Xu Q., Chen M.J., Liao Y., Thiyagarajan M., O’Donnell J., Christensen D.J., Nicholson C., Iliff J.J. (2013). Sleep Drives Metabolite Clearance from the Adult Brain. Science.

[4] Killgore W.D.S., Kerkhof G.A., van Dongen H.P.A. (2010). Progress in Brain Research.

[5] Van Dongen H.P.A., Maislin G., Mullington J.M., Dinges D.F. (2003). The Cumulative Cost of Additional Wakefulness: Dose-Response Effects on Neurobehavioral Functions and Sleep Physiology From Chronic Sleep Restriction and Total Sleep Deprivation. Sleep.

[6] Hastings M., O’Neill J.S., Maywood E.S. (2007). Circadian clocks: regulators of endocrine and metabolic rhythms. J. Endocrinol..

[7] Patke A., Young M.W., Axelrod S. (2020). Molecular mechanisms and physiological importance of circadian rhythms. Nat. Rev. Mol. Cell Biol..

[8] Borbély A.A. (1982). A two process model of sleep regulation. Hum. Neurobiol..

[9] Achermann P., Borbély A.A. (2003). Mathematical models of sleep regulation. Front. Biosci..

[10] Dijk D.J., Beersma D.G., Daan S. (1987). EEG Power Density during Nap Sleep: Reflection of an Hourglass Measuring the Duration of Prior Wakefulness. J. Biol. Rhythms.

[11] Dijk D.J., Brunner D.P., Beersma D.G., Borbély A.A. (1990). Electroencephalogram power density and slow wave sleep as a function of prior waking and circadian phase. Sleep.

[12] Tononi G., Cirelli C. (2003). Sleep and synaptic homeostasis: a hypothesis. Brain Res. Bull..

[13] Tononi G., Cirelli C. (2014). Sleep and the Price of Plasticity: From Synaptic and Cellular Homeostasis to Memory Consolidation and Integration. Neuron.

[14] Esser S.K., Hill S.L., Tononi G. (2007). Sleep Homeostasis and Cortical Synchronization: I. Modeling the Effects of Synaptic Strength on Sleep Slow Waves. Sleep.

[15] Vyazovskiy V.V., Riedner B.A., Cirelli C., Tononi G. (2007). Sleep Homeostasis and Cortical Synchronization: II. A Local Field Potential Study of Sleep Slow Waves in the Rat. Sleep.

[16] Riedner B.A., Vyazovskiy V.V., Huber R., Massimini M., Esser S., Murphy M., Tononi G. (2007). Sleep homeostasis and cortical synchronization: III. A high-density EEG study of sleep slow waves in humans. Sleep.

[17] Finelli L.A., Baumann H., Borbély A.A., Achermann P. (2000). Dual electroencephalogram markers of human sleep homeostasis: Correlation between theta activity in waking and slow-wave activity in sleep. Neuroscience.

[18] Cajochen C., Wyatt J.K., Czeisler C.A., Dijk D.J. (2002). Separation of circadian and wake duration-dependent modulation of EEG activation during wakefulness. Neuroscience.

[19] Strijkstra A.M., Beersma D.G.M., Drayer B., Halbesma N., Daan S. (2003). Subjective sleepiness correlates negatively with global alpha (8-12 Hz) and positively with central frontal theta (4-8 Hz) frequencies in the human resting awake electroencephalogram. Neurosci. Lett..

[20] Aeschbach D., Matthews J.R., Postolache T.T., Jackson M.A., Giesen H.A., Wehr T.A. (1997). Dynamics of the human EEG during prolonged wakefulness: Evidence for frequency-specific circadian and homeostatic influences. Neurosci. Lett..

[21] Åkerstedt T., Fröberg J.E., Friberg Y., Wetterberg L. (1979). Melatonin excretion, body temperature and subjective arousal during 64 hours of sleep deprivation. Psychoneuroendocrinology.

[22] Strogatz S.H., Kronauer R.E., Czeisler C.A. (1987). Circadian pacemaker interferes with sleep onset at specific times each day: Role in insomnia. Am. J. Physiol..

[23] Zeeuw J.d., Wisniewski S., Papakonstantinou A., Bes F., Wahnschaffe A., Zaleska M., Kunz D., Münch M. (2018). The alerting effect of the wake maintenance zone during 40 hours of sleep deprivation. Sci. Rep..

[24] Lavie P. (1997). Melatonin: Role in Gating Nocturnal Rise in Sleep Propensity. J. Biol. Rhythms.

[25] Dijk D.J., Czeisler C.A. (1995). Contribution of the circadian pacemaker and the sleep homeostat to sleep propensity, sleep structure, electroencephalographic slow waves, and sleep spindle activity in humans. J. Neurosci..

[26] Lavie P. (1986). Ultrashort sleep-waking schedule. III. ‘Gates’ and ‘Forbidden zones’ for sleep. Electroencephalogr. Clin. Neurophysiol..

[27] McMahon W.R., Ftouni S., Drummond S.P.A., Maruff P., Lockley S.W., Rajaratnam S.M.W., Anderson C. (2018). The wake maintenance zone shows task dependent changes in cognitive function following one night without sleep. Sleep.

[28] McMahon W.R., Ftouni S., Diep C., Collet J., Lockley S.W., Rajaratnam S.M.W., Maruff P., Drummond S.P.A., Anderson C. (2021). The impact of the wake maintenance zone on attentional capacity, physiological drowsiness, and subjective task demands during sleep deprivation. J. Sleep Res..

[29] Shekleton J.A., Rajaratnam S.M.W., Gooley J.J., Van Reen E., Czeisler C.A., Lockley S.W. (2013). Improved Neurobehavioral Performance during the Wake Maintenance Zone. J. Clin. Sleep Med..

[30] Cohen M.X. (2014).

[31] Donoghue T., Haller M., Peterson E.J., Varma P., Sebastian P., Gao R., Noto T., Lara A.H., Wallis J.D., Knight R.T. (2020). Parameterizing neural power spectra into periodic and aperiodic components. Nat. Neurosci..

[32] Cole S., Voytek B. (2019). Cycle-by-cycle analysis of neural oscillations. J. Neurophysiol..

[33] Aston-Jones G., Cohen J.D. (2005). An integrative theory of locus coeruleus-norepinephrine function: adaptive gain and optimal performance. Annu. Rev. Neurosci..

[34] Joshi S., Li Y., Kalwani R.M., Gold J.I. (2016). Relationships between Pupil Diameter and Neuronal Activity in the Locus Coeruleus, Colliculi, and Cingulate Cortex. Neuron.

[35] Murphy P.R., O’Connell R.G., O’Sullivan M., Robertson I.H., Balsters J.H. (2014). Pupil diameter covaries with BOLD activity in human locus coeruleus. Hum. Brain Mapp..

[36] Lloyd B., de Voogd L.D., Mäki-Marttunen V., Nieuwenhuis S. (2022).

[37] Reimer J., McGinley M.J., Liu Y., Rodenkirch C., Wang Q., McCormick D.A., Tolias A.S. (2016). Pupil fluctuations track rapid changes in adrenergic and cholinergic activity in cortex. Nat. Commun..

[38] Crevits L., Simons B., Wildenbeest J. (2003). Effect of Sleep Deprivation on Saccades and Eyelid Blinking. ENE.

[39] Moller H.J., Kayumov L., Bulmash E.L., Nhan J., Shapiro C.M. (2006). Simulator performance, microsleep episodes, and subjective sleepiness: normative data using convergent methodologies to assess driver drowsiness. J. Psychosom. Res..

[40] Fatt I., Weissman B.A. (2013).

[41] Kwon K.-A., Shipley R.J., Edirisinghe M., Ezra D.G., Rose G., Best S.M., Cameron R.E. (2013). High-speed camera characterization of voluntary eye blinking kinematics. J. R. Soc. Interface.

[42] Hertig-Godeschalk A., Skorucak J., Malafeev A., Achermann P., Mathis J., Schreier D.R. (2020). Microsleep episodes in the borderland between wakefulness and sleep. Sleep.

[43] Ong J.L., Asplund C.L., Chia T.T.Y., Chee M.W.L. (2013). Now You Hear Me, Now You Don’t: Eyelid Closures as an Indicator of Auditory Task Disengagement. Sleep.

[44] Borbély A.A., Daan S., Wirz-Justice A., Deboer T. (2016). The two-process model of sleep regulation: a reappraisal. J. Sleep Res..

[45] Tobler I. (1985). Endogenous sleep substances and sleep regulation.

[46] Tobler I. (1995). Is sleep fundamentally different between mammalian species?. Behav. Brain Res..

[47] Cary B.A., Turrigiano G.G. (2021). Stability of neocortical synapses across sleep and wake states during the critical period in rats. Elife.

[48] Frank M.G. (2011). Erasing synapses in sleep: Is it time to be SHY?. Neural Plast..

[49] Frank M.G. (2013). Why I Am Not SHY: A Reply to Tononi and Cirelli. Neural Plast..

[50] Hung C.S., Sarasso S., Ferrarelli F., Riedner B., Ghilardi M.F., Cirelli C., Tononi G. (2013). Local experience-dependent changes in the wake EEG after prolonged wakefulness. Sleep.

[51] Bernardi G., Siclari F., Yu X., Zennig C., Bellesi M., Ricciardi E., Cirelli C., Ghilardi M.F., Pietrini P., Tononi G. (2015). Neural and behavioral correlates of extended training during sleep deprivation in humans: Evidence for local, task-specific effects. J. Neurosci..

[52] Ly J.Q.M., Gaggioni G., Chellappa S.L., Papachilleos S., Brzozowski A., Borsu C., Rosanova M., Sarasso S., Middleton B., Luxen A. (2016). Circadian regulation of human cortical excitability. Nat. Commun..

[53] Huber R., Mäki H., Rosanova M., Casarotto S., Canali P., Casali A.G., Tononi G., Massimini M. (2013). Human cortical excitability increases with time awake. Cereb. Cortex.

[54] Casali A.G., Gosseries O., Rosanova M., Boly M., Sarasso S., Casali K.R., Casarotto S., Bruno M.-A., Laureys S., Tononi G., Massimini M. (2013). A theoretically based index of consciousness independent of sensory processing and behavior. Sci. Transl. Med..

[55] Massimini M., Ferrarelli F., Huber R., Esser S.K., Singh H., Tononi G. (2005). Neuroscience: Breakdown of cortical effective connectivity during sleep. Science.

[56] Sarasso S., Rosanova M., Casali A.G., Casarotto S., Fecchio M., Boly M., Gosseries O., Tononi G., Laureys S., Massimini M. (2014). Quantifying cortical EEG responses to TMS in (Un)consciousness. Clin. EEG Neurosci..

[57] Aston-Jones G., Chen S., Zhu Y., Oshinsky M.L. (2001). A neural circuit for circadian regulation of arousal. Nat. Neurosci..

[58] Gooley J.J., Chamberlain K., Smith K.A., Khalsa S.B.S., Rajaratnam S.M.W., Van Reen E., Zeitzer J.M., Czeisler C.A., Lockley S.W. (2011). Exposure to Room Light before Bedtime Suppresses Melatonin Onset and Shortens Melatonin Duration in Humans. J. Clin. Endocrinol. Metab..

[59] Joshi S., Gold J.I. (2020). Pupil Size as a Window on Neural Substrates of Cognition. Trends Cogn. Sci..

[60] Ackermann S., Bennett N.C., Oosthuizen M.K. (2020). The effect of varying laboratory conditions on the locomotor activity of the nocturnal Namaqua rock mouse (Micaelamys namaquensis) and the diurnal Four-striped grass mouse (Rhabdomys dilectus). Zoology.

[61] García-Allegue R., Lax P., Madariaga A.M., Madrid J.A. (1999). Locomotor and feeding activity rhythms in a light-entrained diurnal rodent, Octodon degus. Am. J. Physiol..

[62] Refinetti R. (2020). Circadian rhythmicity of body temperature and metabolism. Temperature.

[63] Refinetti R. (1996). Rhythms of body temperature and temperature selection are out of phase in a diurnal rodent, Octodon degus. Physiol. Behav..

[64] Samson D.R., Crittenden A.N., Mabulla I.A., Mabulla A.Z.P., Nunn C.L. (2017). Hadza sleep biology: Evidence for flexible sleep-wake patterns in hunter-gatherers. Am. J. Phys. Anthropol..

[65] Lieberman H.R., Wurtman J.J., Teicher M.H. (1989). Circadian rhythms of activity in healthy young and elderly humans. Neurobiol. Aging.

[66] Campbell S.S., Tobler I. (1984). Animal sleep: A review of sleep duration across phylogeny. Neurosci. Biobehav. Rev..

[67] Samson D.R., Nunn C.L. (2015). Sleep intensity and the evolution of human cognition. Evol. Anthropol..

[68] Fruth B., Tagg N., Stewart F. (2018). Sleep and nesting behavior in primates: A review. Am. J. Phys. Anthropol..

[69] Cajochen C., Knoblauch V., Kräuchi K., Renz C., Wirz-Justice A. (2001). Dynamics of frontal EEG activity, sleepiness and body temperature under high and low sleep pressure. Neuroreport.

[70] Snipes S., Krugliakova E., Meier E., Huber R. (2022). The theta paradox: 4-8 Hz EEG oscillations reflect both sleep pressure and cognitive control. J. Neurosci..

[71] Åkerstedt T., Gillberg M. (1990). Subjective and objective sleepiness in the active individual. Int. J. Neurosci..

[72] Kirschfeld K. (2005). The physical basis of alpha waves in the electroencephalogram and the origin of the “berger effect. Biol. Cybern..

[73] Caldwell J.A., Prazinko B.F., Hall K.K. (2000). The effects of body posture on resting electroencephalographic activity in sleep-deprived subjects. Clin. Neurophysiol..

[74] Wyatt J.K., Ritz-De Cecco A., Czeisler C.A., Dijk D.-J. (1999). Circadian temperature and melatonin rhythms, sleep, and neurobehavioral function in humans living on a 20-h day. Am. J. Physiol..

[75] Delorme A., Makeig S. (2004). EEGLAB: An open source toolbox for analysis of single-trial EEG dynamics including independent component analysis. J. Neurosci. Methods.

[76] Andrillon T., Burns A., Mackay T., Windt J., Tsuchiya N. (2021). Predicting lapses of attention with sleep-like slow waves. Nat. Commun..

[77] Fattinger S., Kurth S., Ringli M., Jenni O.G., Huber R. (2017). Theta waves in children’s waking electroencephalogram resemble local aspects of sleep during wakefulness. Sci. Rep..

[78] Quercia A., Zappasodi F., Committeri G., Ferrara M. (2018). Local Use-Dependent Sleep in Wakefulness Links Performance Errors to Learning. Front. Hum. Neurosci..

[79] Vyazovskiy V.V., Olcese U., Hanlon E.C., Nir Y., Cirelli C., Tononi G. (2011). Local sleep in awake rats. Nature.

[80] Kret M.E., Sjak-Shie E.E. (2019). Preprocessing pupil size data: Guidelines and code. Behav Res.

[81] Hentschke H., Stüttgen M.C. (2011). Computation of measures of effect size for neuroscience data sets. Eur. J. Neurosci..

[82] Cohen J. (1988).

[83] Benjamini Y., Hochberg Y. (1995). Controlling the false discovery rate: a practical and powerful approach to multiple testing. J. Roy. Stat. Soc. B.

