# Supplementary material for: How and when EEG reflects changes in neuronal connectivity due to time awake
Source: iScience. 2023 Jun 19;26(7):107138. doi: 10.1016/j.isci.2023.107138 (PMC10391938; doi:10.1016/j.isci.2023.107138)
Supplement: Document S1. Figures S1–S3 [file mmc1.pdf]

iScience, Volume *26*

Supplemental information

How and when EEG reflects changes in neuronal connectivity due to time awake

Sophia Snipes, Elias Meier, Sarah Nadine Meissner, Hans-Peter Landolt, and Reto Huber

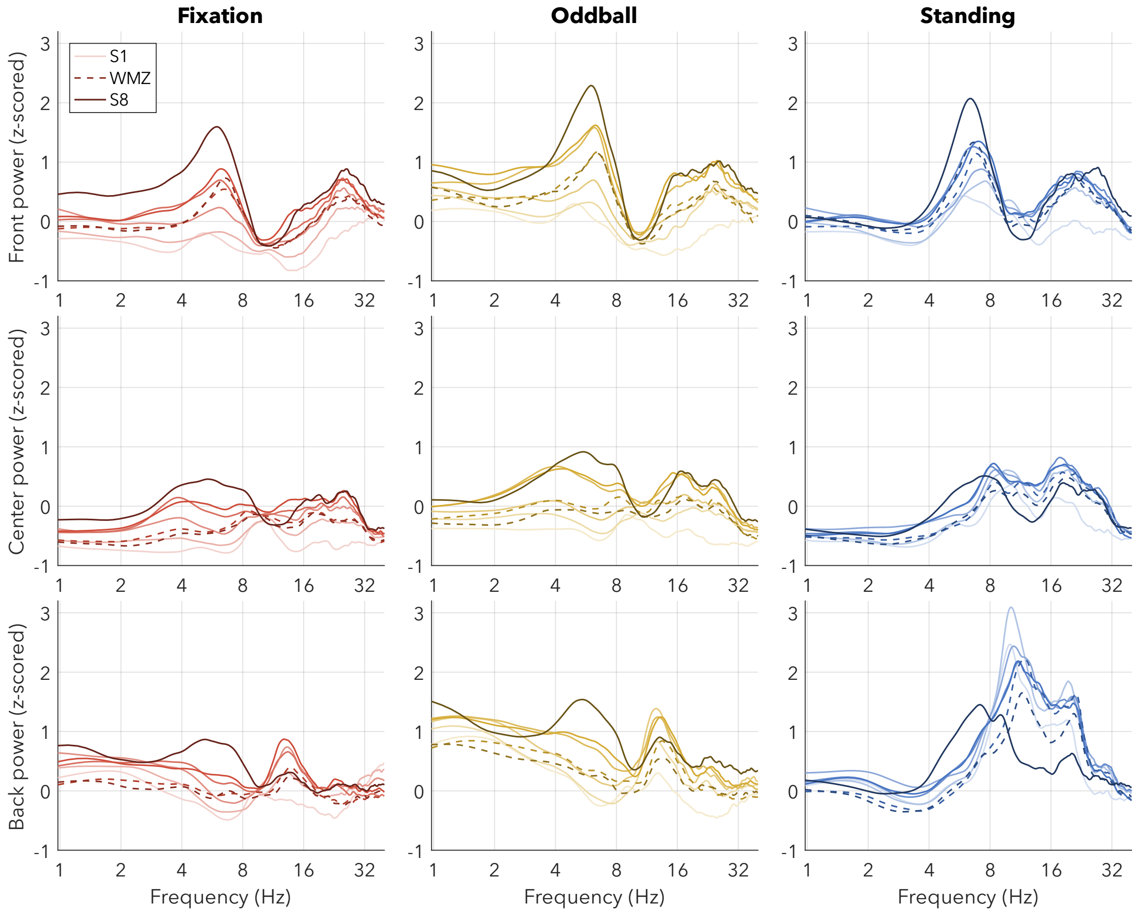

Figure S1. Z-scored power spectrums across extended wake, related to Figure 3. Each row plots an ROI (Front, Center, Back), each column a different condition. Color darkness indicates session, from S1 to S8, such that darker lines indicate more time awake. Dashed lines are the WMZ recordings (S6, S7). The x-axis indicates frequency on a log scale. Acronyms: WMZ, wake maintenance zone; ROI, region of interest.

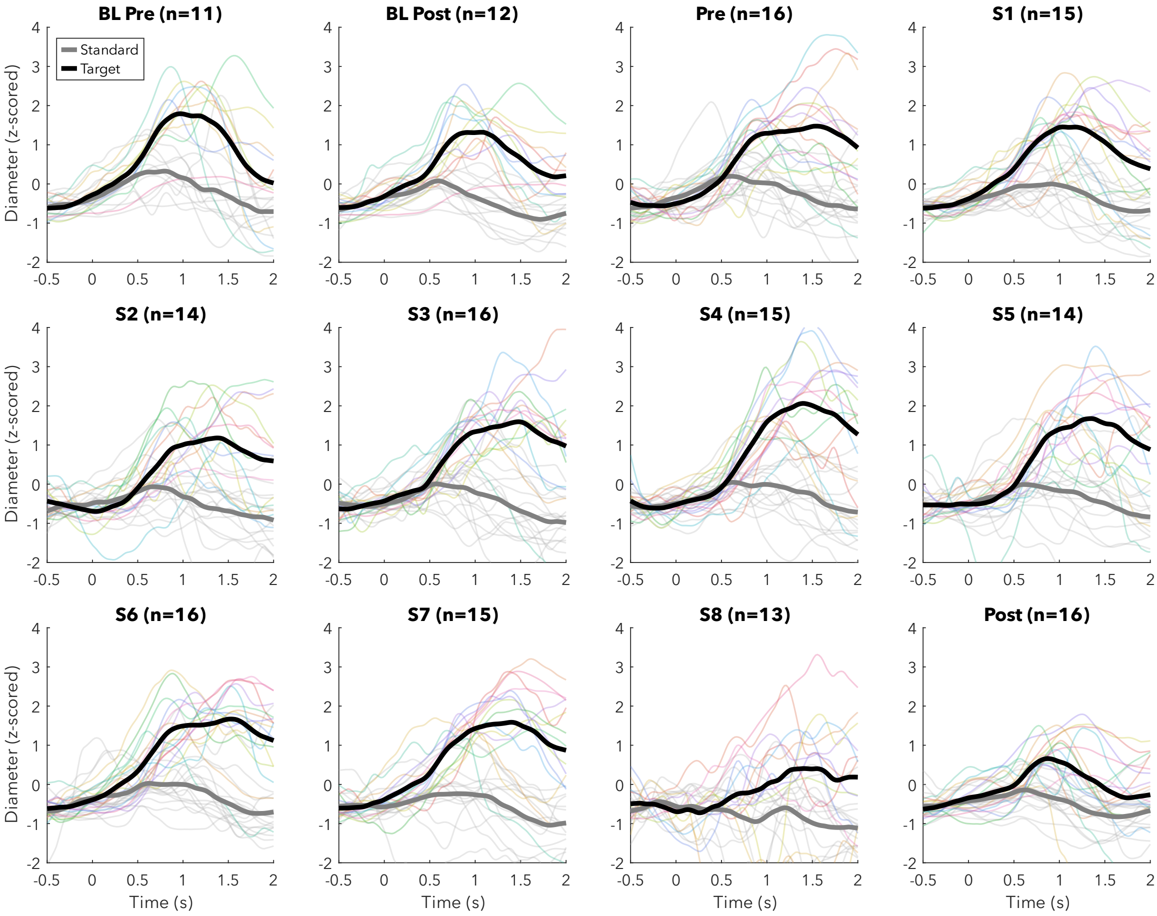

Figure S2. Pupil response to tones in the Oddball, related to Figure 7. Pupil diameters were locked to tone onset, baseline corrected (-.5 to 0 s from tone onset), and then z-scored pooling timepoints, tone type (target and standard), and session. Group average standards are in gray, oddball targets in black. Individuals' average pupil response to standard tones are thin gray lines, and individuals' target responses are thin colored lines. Timecourses were smoothed over 2 s for visualization. Due to data loss and increasing noise, multiple recording sessions were lost, and so the sample size for each session is indicated in the figure titles. Acronyms: BL, baseline.

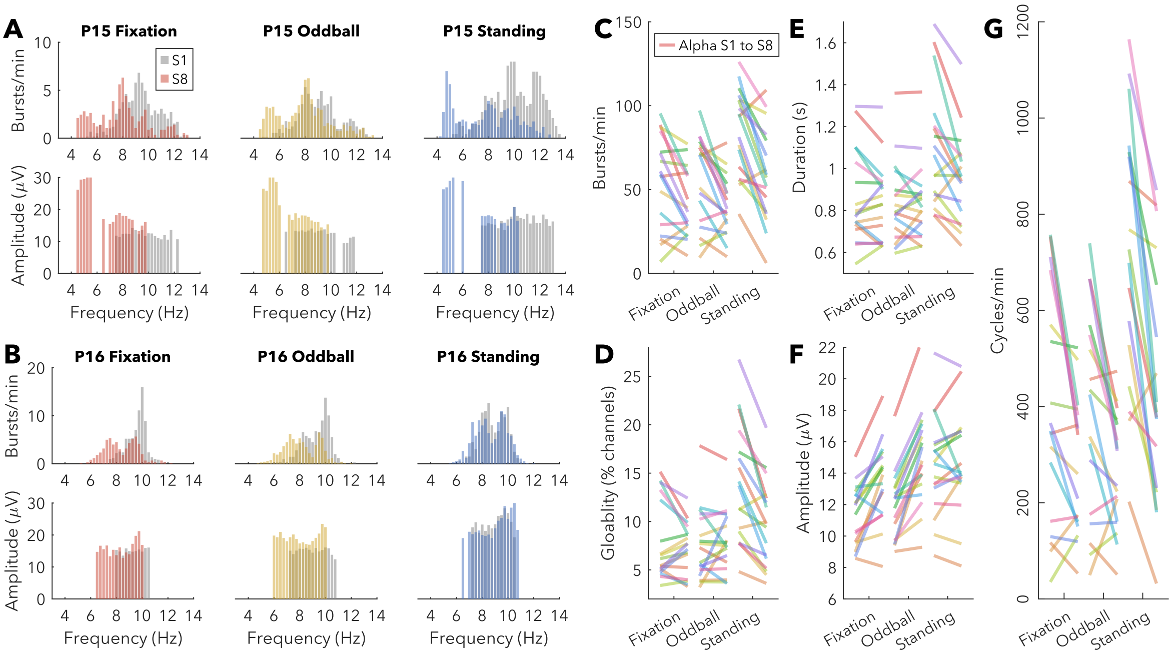

Figure S3. Burst properties, related to STAR Methods. **A-B**) Distribution of number of bursts per minute for each frequency (top plot) and average amplitudes (bottom plot) for two participants. Gray histogram depicts data from the first extended wake recording (S1), and colored histograms the last (S8). Missing values in the amplitude plot correspond to bins for which there were fewer than 10 bursts across the 6 min recording. **C**) Alpha bursts per minute for all participants from S1 (left point of each colored line) to S8 (right point) for each condition. Each participant is a different color. **D**) Average alpha globality, measured as the percentage of channels with an overlapping burst within ±1 Hz of the reference burst. **E**) Average alpha burst duration in seconds. **F**) Average alpha amplitudes, in microvolts. **G**) Average cycles per minute.
